# New Bufadienolides Isolated from the Roots of *Kalanchoe daigremontiana* (Crassulaceae)

**DOI:** 10.3390/molecules21030243

**Published:** 2016-02-24

**Authors:** Barbara Moniuszko-Szajwaj, Łukasz Pecio, Mariusz Kowalczyk, Anna Stochmal

**Affiliations:** Department of Biochemistry and Crop Quality, Institute of Soil Science and Plant Cultivation—State Research Institute, ul. Czartoryskich 8, 24-100 Puławy, Poland; lpecio@iung.pulawy.pl (Ł.P.); mkowalczyk@iung.pulawy.pl (M.K.); asf@iung.pulawy.pl (A.S.)

**Keywords:** *Kalanchoe daigremontiana*, Crassulaceae, bufadienolides, NMR

## Abstract

An aqueous extract from the roots of *Kalanchoe daigremontiana* turned out to be a rich source of bufadienolides. The existing literature data relate mainly to the aerial parts of *Kalanchoe* but there is no information about the metabolic profile of the roots, which are also used in traditional medicine. Our investigation concerning the roots of *K. daigremontiana* led to the isolation and characterization of eight new bufadienolides, namely 1β,3β,5β,14β,19-pentahydroxybufa-20,22-dienolide (**1**), 19-(acetyloxy)-1β,3β,5β,14β-tetrahydroxybufa-20,22-dienolide (**2**), 3β-*O*-α-l-rhamno-pyranosyl-5β,11α,14β,19-tetrahydroxybufa-20,22-dienolide (**3**), 19-(acetyloxy)-3β,5β,11α,14β-tetrahydroxybufa-20,22-dienolide (**4**), 3β,5β,11*α*,14β,19-pentahydroxy-12-oxo-bufa-20,22-dienolide (**5**), 19-(acetyloxy)-3β,5β,11α,14β-tetrahydroxy-12-oxo-bufa-20,22-dienolide (**6**), 19-(acetyloxy)-1β,3β,5β,11α,14β-pentahydroxy-12-oxo-bufa-20,22-dienolide (**7**) and 1β-(acetyloxy)-3β,5β,11*α*,14β,19-pentahydroxy-12-oxo-bufa-20,22-dienolide (**8**), together with seven known compounds: 11α,19-dihydroxytelocinobufagin (**9**), bersaldegenin-1-acetate (**10**), daigredorigenin-3-acetate (**11**), bersaldegenin-1,3,5-orthoacetate (**12**), bryotoxin B (**13**), bryophyllin B (**14**) and bersaldegenin (**15**). The structures were established applying extensive 1D- and 2D-NMR and MS spectroscopic analyses.

## 1. Introduction

*Kalanchoe daigremontiana* (*Bryophyllum daigremontianum*) Raym.-Hamet & H. Perrier (common name: Mother of Thousands), from the Crassulaceae family, is a succulent herb native to the dry zones of Madagascar [1]. *K. daigremontiana* is used as an alternative treatment for skin wounds, arthritis, and gastric ulcers [2]. Saps of different species of genus *Kalanchoe* demonstrate antifungal [3], antihistaminic [4], immunosuppressive [5,6], antileishmanial [7,8], insecticidal [9,10], antitumor [11], hepatoprotective [12], and anti-inflammatory activities [13]. Some species of the *Kalanchoe* genus, like *Kalanchoe daigremontiana* × *tubiflora*, have been used in folk medicine for a long time for the treatment of many diseases, including infections, rheumatism, cough, fever, and inflammation [10,14,15].

Bufadienolides belong to the cardiac glycosides, which are naturally occurring C-24 steroids bearing a characteristic α-pyrone ring at C-17, with a powerful stimulating action on the cardiac muscle. They have been isolated from materials of both animal and plant origin [16,17]. Until now, bufadienolides have been found in six plant families: Crassullaceae, Scilloideae, Iridaceae, Melianthaceae, Ranunculaceae and Santalaceae. The efficacy of these substances was already known in ancient Egypt where squill (Scilloideae) was used in the treatment of heart diseases. Scillaren A, one of the main glycosides of squill, was the first identified bufadienolide, the structure of which was elucidated in 1933 [16].

Bufadienolides are known to have the following biological activities: positive inotropic, and central nervous system effect, local anesthetic, sedative, and cytotoxic. The cytotoxic activity of a series of naturally occurring bufadienolides has been studied through determining their anti-tumor activity toward several cell lines, including KB cells, human lung carcinoma A-549, colon HCT-8 tumor cells, human carcinoma of the nasopharynx, Walker intramuscular 256 carcinosarcoma, HeLa cell line, Bel-7420 cell line, HL-60 cell line, astrocytoma, prostate carcinoma and gastric tumor cell line [11,17,18].

Preliminary research of the roots showed the presence of phenolic compounds and a considerable amount of bufadienolides, with structures probably different from those found in the aerial parts. The aim of this work was to isolate individual compounds from the roots of *Kalanchoe daigremontiana* and determine their structure.

## 2. Results and Discussion

**Figure 1:**
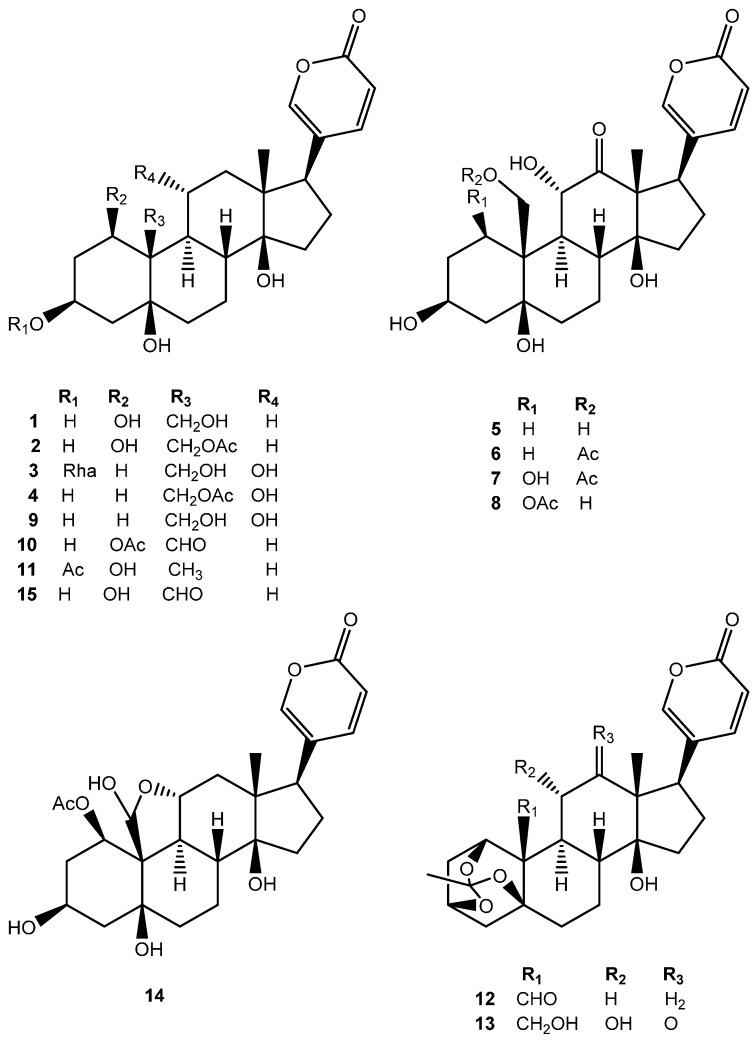
Chemical structures of compounds **1**–**15**.

The H_2_O extract of the *Kalanchoe daigremontiana* roots was successively subjected to a column chromatography over a reverse-phase C_18_ silica gel, Sephadex LH-20 and semi-preparative HPLC to produce 15 bufadienolides **1**–**15** (Figure 1).

**Figure 2:**
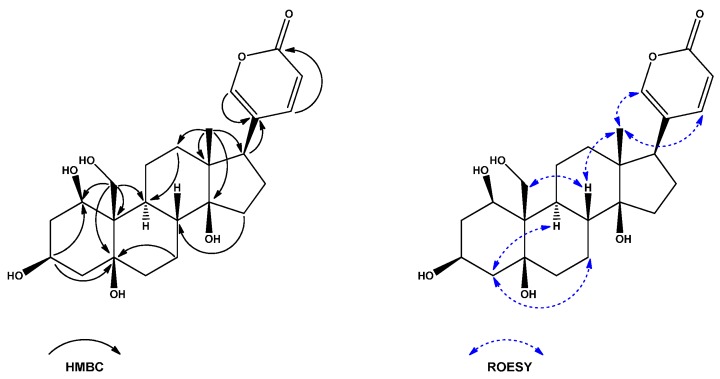
Key HMBC and ROESY correlations of **1**.

Compound **1** was obtained as a white powder with ${\left[\alpha \right]}_{D}^{20}$ = −23.840° (*c* 0.25, MeOH). The quasi-molecular ion at *m*/*z* 433.2235 [M − H]^−^ in its HR-ESI-TOF-MS suggested that the molecular formula of **1** was C_24_H_34_O_7_ and indicated eight degrees of unsaturation. Its IR spectrum indicated the presence of hydroxyl (3423 cm^−1^), carbonyl (1714 cm^−1^), and olefin (1631 cm^−1^) functionalities. The UV spectrum implied the presence of a 2*H*-pyran-2-one moiety (λ_max_ 299 nm). In the ^1^H-NMR spectrum of **1**, a signal for one angular methyl [δ_H_ 0.74 (3H, s)], one hydroxymethylene [δ_H_ 4.06 (1H, d, *J* = 11.6 Hz) and δ_H_ 4.40 (1H, d, *J* = 11.8 Hz)] and a 2*H*-pyran-2-one unit [δ_H_ 6.28 (1H, dd, *J* = 9.7, 1.1 Hz), 7.42 (1H, dd, *J* = 2.6, 1.1 Hz), and 7.98 (1H, dd, *J* = 9.7, 2.6 Hz)] were observed. Analysis of ^13^C-NMR and DEPT spectra revealed that **1** possessed 24 carbon atoms, composed of six quaternary carbons, eight methines, nine methylenes and one methyl. The carbon signals at δ_C_ 115.5 (C-23), 125.0 (C-20), 149.3 (C-22), 150.5 (C-21) and 164.8 (C-24) were typical for a 2*H*-pyran-2-one moiety. This evidence indicated that **1** possessed a bufadienolide skeleton. All the ^13^C- and ^1^H-NMR signals (Table 1 and Table 2) of **1** were assigned unambiguously by an extensive analysis of ^1^H-^1^H COSY, HSQC, H2BC, HSQC-TOCSY and HMBC data. The HMBC correlation between H_2_-19 (δ_H_ 4.06 and 4.40) and C-5 (δ_C_ 76.5) suggested the existence of 5-OH, and correlation between Me-18 (δ_H_ 0.74) and C-14 (δ_C_ 85.9) suggested the existence of 14-OH. Furthermore, the correlation between H_2_-19 and C-1 (δ_C_ 71.7) indicated that the hydroxyl group was also located at C-1 (Figure 2). It was supported by the correlation visible in the HSQC spectrum between C-1 and H-1 (δ_H_ 4.42, dd, *J* = 3.3, 3.3 Hz). Small coupling constants measured between H-1 and both H-2α (δ_H_ 1.98, ddd, *J* = 14.9, 3.5, 3.5) and H-2β (δ_H_ 2.08, dddd, *J* = 15.0, 2.5, 2.5, 2.5 Hz) suggested that the hydroxyl group at C-1 should be β-oriented. This was further supported by the NOE effect visible in the ROESY spectra between H-1 and H-11α (δ_H_ 1.51, m). Additionally, the correlation visible in the HMBC spectra between H-1 and C-3 (δ_C_ 68.1), suggested that **1** was a 1,3,5,14,19-pentahydroxybufadienolide. The NOE correlations observed in the ROESY spectrum between H-8 (δ_H_ 1.80), Me-18 and H-19 suggested their 1,3-diaxial configuration and β-orientation. The cross-peaks in ROESY spectra between H-4α (δ_H_ 2.32)/H-2α (δ_H_ 1.98) and H-9 (δ_H_ 1.46) indicated that these protons were *α*-oriented. Additionally, the NOE correlations between Me-18 (δ_H_ 0.74) and H-21 (δ_H_ 7.42)/H-22 (δ_H_ 7.98) confirmed the β-orientation of the 2*H*-pyran-2-one unit (Figure 2) Therefore, A/B *cis* and B/C *trans* ring junctures was deduced. Furthermore, in order to fully recognize the junction between ring C/D of **1**, NMR spectra were additionally acquired in deuterated DMSO (Table S1, Supplementary Materials). In ROESY spectra acquired in DMSO-*d*_6_ NOE enhancement between Me-18 (δ_H_ 0.62, s) and 14-OH (δ_H_ 4.16, s) suggested that the relative configuration of 14-OH was β, indicating C/D *cis* ring juncture, which was in line with the biogenesis pathway. However, to be more precise, Electronic Circular Dichroism (ECD) spectra were acquired (Figures S9–S16, Supplementary Materials) and Cotton effects presented by compounds **1**–**8** were similar with these of known bufadienolides [19], consequently confirming the relative stereochemistry of compounds **1**–**8**. Thus, the structure of **1** was established as 1β,3β,5β,14β,19-pentahydroxybufa-20,22-dienolide.

Compound **2** was isolated as a white powder. The molecular formula of **2** was determined to be C_26_H_36_O_8_ by its HR-ESI-TOF-MS data (*m*/*z* 475.2338 [M − H]^−^; calcd for C_26_H_35_O_8_: 475.2337). The UV spectrum indicated the presence of a 2*H*-pyran-2-one moiety. Comparison of the ^1^H- and ^13^C-NMR data of **2** with those of **1** revealed their structural similarity, except for the presence of the signals for a methyl [δ_H_ 2.05 (3H, s), δ_C_ 21.3] and carbonyl (δ_C_ 173.0) group in **2**, characteristic of an acetoxy group. It was supported by a long-range correlation visible in the HMBC spectrum between downfield shifted H_2_-19 (δ_H_ 4.62 and 4.81) and the carbonyl group. Additionally, γ-effect was observed for C-5 (δ_C_ 75.3), which was shifted upfield by Δδ 1.2 ppm comparing to **1**. In order to confirm relative stereochemistry of **2** ROESY spectra were acquired and presented similar to compound **1** NOE enhancements. Based on these results, the structure of **2** was deduced to be 19-(acetyloxy)-1β,3β,5β,14β-tetrahydroxybufa-20,22-dienolide.

Compound **3** was obtained as a white powder. The molecular formula of **3** was determined to be C_30_H_44_O_11_ by its HR-ESI-TOF-MS data (*m*/*z* 579.2808 [M − H]^−^; calcd for C_30_H_43_O_11_: 579.2811). The UV spectrum indicated the presence of a 2*H*-pyran-2-one moiety. The ^1^H- and ^13^C-NMR spectra of the genin part of **3** was very similar to that of 11α,19-dihydroxytelocinobufagin (3β,5β,11α,14,19-pentahydroxybufa-20,22-dienolide, **9**, Tables S2 and S3—Supplementary Materials), which was also isolated from this plant. In addition, compound **3** had one sugar unit attached to the genin, which was confirmed by the presence of one anomeric proton at δ_H_/δ_C_ 4.85/100.9. The glycosyl moiety was identified as α-rhamnopyranoside by a three-proton doublet signal at δ 1.26 (3H, d, *J* = 6.3 Hz, H-6′), which was correlated in HSQC spectra with the methyl carbon signal at δ 18.0 (C-6′) and five other oxymethine signals in TOCSY spectra at δ_H_ 3.40 (1H, dd, *J* = 9.5, 9.5 Hz, H-4′), 3.63 (1H, dd, *J* = 9.5, 3.5 Hz, H-3′), 3.65 (1H, dq, *J* = 9.5, 6.2 Hz, H-5′), 3.78 (1H, dd, *J* = 3.3, 1.7 Hz, H-2′) and δ_C_ 70.6 (C-5′), 72.5 (C-3′), 72.6 (C-2′), 73.8 (C-4′). The α-orientation of an anomeric proton was confirmed by the ^1^*J*_CH_ coupling constant (168 Hz). An HMBC correlation between H-1′ (δ_H_ 4.85) and C-3 (δ_C_ 76.0) and a NOE correlation visible in the ROESY spectra between H-1′ and H-3 (δ_H_ 4.15) established that the glycosidic moiety was linked to C-3 of the aglycone. The structure of **3** was thus established as 3β-(O-α-l-rhamnopyranosyl)-5β,11α,14β,19-tetrahydroxybufa-20,22-dienolide.

Compound **4** was obtained as a white powder. The molecular formula of **4** was determined to be C_26_H_36_O_8_ by its HR-ESI-TOF-MS data (*m*/*z* 475.2339 [M − H]^−^; calcd for C_26_H_35_O_8_: 475.2337). The UV spectrum indicated the presence of a 2*H*-pyran-2-one moiety. Comparison of the ^1^H- and ^13^C-NMR data of **4** with those of **9** revealed their structural similarity, except for the presence of the signals for a methyl [δ_H_ 2.07 (3H, s), δ_C_ 21.2] and carbonyl (δ_C_ 173.0) group in **4**, characteristic for an acetoxy group. It was supported by a long-range correlation visible in the HMBC spectrum between downfield shifted H_2_-19 (δ_H_ 4.45 and 4.42) and the carbonyl group. Additionally, γ-effect was observed for C-5 (δ_C_ 75.9), which was shifted upfield by Δδ 2.8 ppm comparing to **9**. Based on these results, the structure of **4** was deduced to be 19-(acetyloxy)-3β,5β,11α,14β-tetrahydroxybufa-20,22-dienolide.

Compound **5** was obtained as a white powder. The molecular formula of **5** was determined to be C_24_H_32_O_8_ by its HR-ESI-TOF-MS data (*m*/*z* 447.2021 [M − H]^−^: calcd for C_24_H_31_O_8_: 447.2024). The UV spectrum indicated the presence of a 2*H*-pyran-2-one moiety. Comparison of the ^1^H- and ^13^C-NMR spectra of **5** with those of a known group of compounds—the lucibufagins [20,21], suggested their structural similarity, except for the presence of the signals for an oxygenated methylene [δ_H_ 4.01 (1H, d, *J* = 11.3 Hz) and 4.19 (1H, d, *J* = 11.3 Hz), δ_C_ 65.9] instead of a methyl group. Thus, **5** was proposed to be a lucibufagin derivative. In the HMBC spectrum of **5**, the correlation between H_2_-19 (δ_H_ 4.01 and 4.19) and C-1 (δ_C_ 21.9)/C-9 (δ_C_ 43.9)/C-10 (δ_C_ 45.4)/C-5 (δ_C_ 78.1) suggested the methylene group was located at C-19. Furthermore, the correlations between Me-18 (δ_H_ 0.92)/H-9 (δ_H_ 1.80) and C-12 (δ_C_ 214.5) indicated that *oxo*- group was located at C-12. It was supported by the downfield shift of H-11/C-11 at δ_H_/δ_C_ 4.71/75.1, downfield shift of C-13 at δ_C_ 63.6, downfield shift of H-17 at δ_H_ 4.11, and γ-effect observed for C-17 (δ_C_ 42.1), which was shifted upfield by Δδ 10 ppm comparing to **1**–**4** (Table 1 and Table 3). Based on these results, the structure of **5** was deduced to be3β,5β,11α,19-pentahydroxy-12-oxo-bufa-20,22-dienolide.

Compound **6** was obtained as a white powder. The molecular formula of **6** was determined to be C_26_H_34_O_9_ by its HR-ESI-TOF-MS data (*m*/*z* 489.2125 [M − H]^−^; calcd for C_26_H_33_O_9_: 489.2130). The UV spectrum indicated the presence of a 2*H*-pyran-2-one moiety. Comparison of the ^1^H- and ^13^C-NMR data of **6** with those of **5** revealed their structural similarity, except for the presence of the signals for a methyl [δ_H_ 2.08 (3H, s), δ_C_ 21.2] and carbonyl (δ_C_ 172.8) group in **6**, characteristic for an acetoxy group. It was supported by a long-range correlation visible in the HMBC spectrum between downfield shifted H_2_-19 (δ_H_ 4.50 and 4.60) and the carbonyl group. Additionally, γ-effect was observed for C-5 (δ_C_ 75.9), which was shifted upfield by Δδ 2.2 ppm comparing to **5**. Based on these results, the structure of **6** was deduced to be 19-(acetyloxy)-3β,5β,11α,14β-tetrahydroxy-12-oxo-bufa-20,22-dienolide.

Compound **7** was obtained as a white powder. The molecular formula of **7** was determined to be C_26_H_34_O_10_ by its HR-ESI-TOF-MS data (*m*/*z* 505.2090 [M − H]^−^; calcd for C_26_H_33_O_10_: 505.2079). The UV spectrum indicated the presence of a 2*H*-pyran-2-one moiety. The NMR spectroscopic features of **7** were similar to the compound **6**. A detailed examination of 1D and 2D NMR data of **7** revealed that a methylene group located at C-1 in **6** was replaced by an oxygenated methine (δ_H_ 5.24, δ_C_ 71.0). In the ^1^H-^1^H COSY spectrum of **7**, the methine proton (δ_H_ 5.24, H-1) was correlated with H-2*α* (δ_H_ 2.14) and H-2β (δ_H_ 2.04), indicating that a hydroxyl group was attached to C-1. It was further supported by a correlation visible in the HMBC spectra between H_2_-19 (δ_H_ 4.85 and 5.00) and C-1 (δ_C_ 71.0). Appearance of H-1 as a broad singlet was observed, suggesting that the hydroxyl group at C-1 was β-oriented. Thus the structure of **7** was established as 19-(acetyloxy)-1β,3β,5β,11α,14β-pentahydroxy-12-oxo-bufa-20,22-dienolide.

Compound **8** was obtained as a white powder. The molecular formula of **8** was determined to be C_26_H_34_O_10_ by its HR-ESI-TOF-MS data (*m*/*z* 505.2088 [M − H]^−^; calcd for C_26_H_33_O_10_: 505.2079), which was the same as **7**. The UV spectrum indicated the presence of a 2*H*-pyran-2-one moiety. Comparison of the ^1^H- and ^13^C-NMR spectra of **8** and **7** implied that these two compounds were almost the same, and differed only in the position of an acetoxy group. According to the HMBC spectral analysis, correlation between downfield shifted H-1 (δ_H_ 6.52) and a carbonyl group at δ_C_ 172.6, indicated the location of the acetoxy group at C-1 (δ_C_ 74.1). It was further supported by the correlation visible between shifted upfield H_2_-19 (δ_H_ 4.29 and 4.37) and C-9 (δ_C_ 46.5)/C-10 (δ_C_ 49.3)/C-1 (δ_C_ 74.1). The relative stereochemistry of **8** was determined to be the same as **7** based on the ROESY spectral analysis. The NOE correlations between H-8 (δ_H_ 2.49), Me-18 (δ_H_ 0.95) and H_2_-19, suggested their 1,3-diaxial configuration and β-orientation, as in **7**. Thus, the structure of compound **8** was elucidated as 1β-(acetyloxy)-3β,5β,11α,14β,19-pentahydroxy-12-oxo-bufa-20,22-dienolide.

There are only few articles describing bufadienolides found in *Kalanchoe daigremontiana*. Wagner isolated bersaldegenin-1,3,5-orthoacetate, bersaldegenin-1-acetate, daigredorigenin-3-acetate and daigremontianin [22] and Supratman isolated the same compounds as well as bryophyllin A and C, bersaldegenin-3-acetate and methyl daigremonate from *Kalanchoe daigremontiana* × *tubiflora,* a hybrid plant between *K. daigremontiana* and *K. tubiflora* [10].

## 3. Experimental Section

### 3.1. General Information

Optical rotations were measured by using an automatic digital polarimeter (P-2000, Jasco, Tokyo, Japan). IR spectra were recorded on a FT-IR 6200 spectrophotometer (Jasco). The circular dichroism spectra were obtained on a Chirascan-plus CD Spectrometer (Applied Photophysics Limited, Leatherhead, Surrey, UK). The ESI-MS/MS analyses were carried out according to the previously developed procedures [23] on an ACQUITY UPLC™ system (Waters, Milford, MA, USA), equipped with a PDA and a triple quadrupole mass detector (ACQUITY TQD, Waters). Samples were separated on a BEH C18 (100 × 2.1 mm, 1.7 µm; ACQUITY, Waters) column, maintained at 40 °C. The elution (400 µL·min^−1^) was carried out with a gradient of solvent B (acetonitrile with 0.1% formic acid) in solvent A (water with 0.1% formic acid): 0–1 min, 5% B; 1–24 min 5%–50% B; 24–25 min 50%–95% B; 25–27 min 95% B; 27–27.1 min, 95%–5% B; 27.1–30 min, 5% B. Mass analyses were performed in the negative ionization mode (scan *m*/*z* = 300–1100). The capillary and cone voltages were at 2.8 kV and 40 V, respectively; source and desolvation temperatures were kept at 140 °C and 350 °C, respectively. Nitrogen was used as cone and desolvation gas at 100 L·h^−1^ and 800 L·h^−1^, respectively. The collision gas (argon) flow was 0.1 mL·min^−1^. Exact masses of isolated bufadienolides were determined by high resolution LC-MS (HR-ESI-MS) analyses which were performed with the Thermo Ultimate 3000 RS (Thermo Fischer Scientific, Waltham, MS, USA) chromatographic system coupled with a Bruker Impact II HD (Bruker, Billerica, MA, USA) quadrupole-time of flight (Q-TOF) mass spectrometer. Chromatographic separations were carried out on a Waters BEH C18 column (100 × 2.1 mm, 1.7 µm, Milford, MA, USA). The mobile phase A was 0.1% (*v*/*v*) formic acid, and the mobile phase B was acetonitrile containing 0.1% (*v*/*v*) of formic acid. A linear gradient from 5% of phase B to 60% of phase B over 22 min was used to separate analytes. The flow rate was 400 µL·min^−1^ and the column was kept at 35 °C. Between the injections, the column was equilibrated with 10 volumes of 5% phase B. All data were acquired using electrospray ionization in the negative ion mode. Linear (centroid) spectra were acquired over a mass range from *m*/*z* 100 to *m*/*z* 2000 with the following parameters of the mass spectrometer: capillary voltage 3 kV; dry gas flow 4 L·min^−1^; dry gas temperature 180 °C; nebulizer pressure 0.4 bar; collision RF 800.0 Vpp; transfer time 100.0 μs; prepulse storage 5.0 μs. Collision energy was set automatically from the range of 20 to 120 eV, depending on the *m*/*z* of a fragmented ion. Acquired data were calibrated internally with a sodium formate introduced to the ion source via a 20 µL loop at the beginning of each separation. NMR spectra were acquired in CD_3_OD or DMSO-*d*_6_ at 25 °C on an Avance III HD 500 MHz instrument (^1^H: 500.18 MHz; ^13^C 125.79 MHz; Bruker BioSpin, Rheinstetten, Germany) equipped with an inverse BBI probe. Standard pulse sequences and parameters were used to obtain 1D ^1^H, 1D selective TOCSY, 1D selective ROESY, 1D ^13^C and DEPT-135, gCOSY, TOCSY (mixing time 120 ms), ROESY (mixing time 250 ms), gHSQC, gHSQC-TOCSY (mixing time 120 ms), gH2BC and gHMBC spectra. Chemical shift referencing was carried out using the internal solvent resonances at δ_H_ 3.31 and δ_C_ 49.0 (CD_3_OD), or at δ_H_ 2.50 and δ_C_ 39.51 (DMSO-*d*_6_), calibrated to TMS at 0.00 ppm. Chemical shifts are in ppm and the *J* values in Hz. Data processing was performed with the Topspin software (version 3.5pl2, Bruker BioSpin). Analytical Thin Layer Chromatography (TLC) was carried out on precoated silica gel 60 F_254_ TLC plates (art 105554, Merck, Darmstadt, Germany) using 60:15:20:5 MeCN/H_2_O/CHCl_3_/HCOOH solvent system. The plates were visualized under ultraviolet light (λ_254_ nm) and by charring the compounds with 5% sulphuric acid in MeOH. A vacuum liquid chromatography was carried out on LiChroprep RP-18 (40–63 μm) (Merck). A low-pressure column chromatography was done using Sephadex LH-20 (25–100 μm, Sigma-Aldrich, St. Luis, MO, USA) and LiChroprep RP-18 (40–63 µm, Merck). Semi-preparative HPLC was performed on an Analytical to Semi-preparative HPLC system (Gilson Inc., Middleton, WI, USA) equipped with an Evaporative Light Scattering—PrepELS II (ELSD) detector (Gilson Inc.). The drift tube of ELSD detector was maintained at 65 °C and the pressure of the nebulizer gas—nitrogen—was 47 psi. The outgoing effluent from HPLC system was diverted through a passive splitter to ELSD with a split ratio 1:100.

### 3.2. Plant Material

Roots of *Kalanchoe daigremontiana* Raym.-Hamet & H. Perrier were purchased from a commercial source (Horticultural Centre in Wrocław, Poland). A voucher specimen (IUNG/KDR/2012/1) has been deposited at the Department of Biochemistry and Crop Quality, Institute of Soil Science and Plant Cultivation, State Research Institute, Puławy, Poland.

### 3.3. Extraction and Isolation

The ground roots (500 g) of *K. daigremontiana* were extracted three times with water at room temperature for 24 h with an aid of ultrasonic bath. After each extraction, the crude extract was filtered through a G2 Büchner funnel. The combined aqueous extracts were concentrated under reduced pressure and lyophilized to yield brown residue (34.8 g). This extract was applied to a vacuum liquid chromatography on LiChroprep RP-18 (100 × 60 mm, 40–63 μm) with H_2_O:MeOH (cont. 0.1% HCOOH) in a stepwise gradient manner to obtain a bufadienolide-rich 80% MeOH fraction (5.6 g). This fraction was further partitioned between water and butanol to afford *n*-BuOH extract (4.6 g), which was applied on a Sephadex LH-20 column (950 × 22 mm, 25–100 μm), eluted with MeOH, and afforded seven distinctive subfractions (F1–F7). Fractions F2 (0.90 g), F3 (1.22 g) and F4 (1.35 g) were separated on a Lichroprep RP-18 column (32 × 320 mm, 40–63 μm), eluted with a linear gradient of 15%–40% aqueous MeOH containing 0.1% HCOOH. Individual bufadienolides were further purified by a reversed-phase semi-preparative HPLC isocratically on a C18 column (Kromasil 100-5-C18, 250 × 10 mm, 5 μm), using aqueous methanol solutions of different concentrations (ranging from 25% to 50% MeOH), and containing 0.1% HCOOH. The mobile phase flow was maintained at 4 mL·min^−1^, and the column temperature was maintained at 30 °C. Fraction F2 yielded compound **3** (11 mg); fraction F3 yielded compounds **1** (5.3 mg), **2** (11.2 mg), **4** (6.1 mg), **5** (15.4 mg), **9** (25 mg), **10** (6 mg), **11** (9.5 mg), **13** (6.4 mg), **14** (8.5 mg) and **15** (6.2 mg); fraction F4 yielded compounds **6** (14.7 mg), **7** (4.8 mg), **8** (4.6 mg), and **12** (25.8 mg). The structure elucidation of the isolated compounds was performed using optical rotation, ECD, IR, UV, ESI-MS and NMR spectral data analyses and comparison with appropriate literature values.

*1β,3β,5β,14β,19-Pentahydroxybufa-20,22-dienolide* (**1**): White powder (5.3 mg); ${\left[\alpha \right]}_{D}^{20}$ = −23.840° (*c* 0.25, MeOH); ECD (*c* 2.30 × 10^−4^ M, MeOH) λ_max_ (Δε) 222 (+1.48), 250 (-0.43), 293 (+0.31); IR υ_max_: 3423 (OH), 2939 and 2878 (CH), 1714 (C=O), 1631 (C=C), 1539, 1247, 1123, 1080, 1035 cm^−1^; UV (UPLC-PDA) λ_max_ (nm): 299; HR-ESI-TOF-MS, *m*/*z*: 433.2235 [M − H]^−^ (calc. for C_24_H_33_O_7_ = 433.2232); ESI-MS/MS (TOF), *m*/*z*: 479 [M − H]^−^, 433 [M − H − 46]^−^; ^13^C- and ^1^H-NMR data are shown in Table 1 and Table 2, respectively.

*19-(Acetyloxy)-1β,3β,5β,14β-tetrahydroxybufa-20,22-dienolide* (**2**): White powder (11.2 mg); ${\left[\alpha \right]}_{D}^{20}$ = −12.287° (*c* 0.39, MeOH); ECD (*c* 2.10 × 10^−4^ M, MeOH) λ_max_ (Δε) 220 (+1.90), 247 (-0.83), 300 (+0.46); IR υ_max_: 3430 (OH), 2945 and 2878 (CH), 1715 (C=O), 1632 (C=C), 1539, 1247, 1124, 1083, 1038 cm^−1^; UV (UPLC-PDA) λ_max_ (nm): 299; HR-ESI-TOF-MS, *m*/*z*: 475.2338 [M − H]^−^ (calc. for C_26_H_35_O_8_ = 475.2337); ESI-MS/MS (TOF), *m*/*z*: 521 [M − H]^−^, 475 [M − H − 46]^−^, 433 [M − H − 46 − 42]^−^; ^13^C- and ^1^H-NMR data are shown in Table 1 and Table 2, respectively.

*3β-(O-α-l-Rhamnopyranosyl)-5β,11α,14β,19-tetrahydroxybufa-20,22-dienolide* (**3**): White powder (11 mg); ${\left[\alpha \right]}_{D}^{20}$ = −36.356° (*c* 0.27, MeOH); ECD (*c* 1.72 × 10^−4^ M, MeOH) λ_max_ (Δε) 220 (+2.39), 249 (−0.75), 300 (+0.22); IR υ_max_: 3413 (OH), 2934 (CH), 1705 (C=O), 1631 (C=C), 1539, 1126, 1037 cm^−1^; UV (UPLC-PDA) λ_max_ (nm): 297; HR-ESI-TOF-MS, *m*/*z*: 579.2808 [M − H]^−^ (calc. for C_30_H_43_O_11_ = 579.2811); ESI-MS/MS (TOF), *m*/*z*: 625 [M − H]^−^, 579 [M − H − 46]^−^ 433 [M − H − 46 − 146]^−^; ^13^C- and ^1^H-NMR data are shown in Table 1 and Table 2, respectively.

*19-(Acetyloxy)-3β,5β,11α,14β-tetrahydroxybufa-20,22-dienolide* (**4**): White powder (6.1 mg); ${\left[\alpha \right]}_{D}^{20}$ = +3.2800° (*c* 0.30, MeOH); ECD (*c* 2.10 × 10^−4^ M, MeOH) λ_max_ (Δε) 225 (+1.25), 251 (−0.25), 279 (+0.41), 300 (+0.25); IR υ_max_: 3412 (OH), 2932 and 2876 (CH), 1712 (C=O), 1630 (C=C), 1251, 1125, 1037 cm^−1^; UV (UPLC-PDA) λ_max_ (nm): 299; HR-ESI-TOF-MS, *m*/*z*: 475.2339 [M − H]^−^ (calc. for C_26_H_35_O_8_ = 475.2337); ESI-MS/MS (TOF), *m*/*z*: 521 [M − H]^−^, 475 [M − H − 46]^−^, 433 [M − H − 46 − 42]^−^, 415 [M − H − 46 − 60]^−^; ^13^C- and ^1^H-NMR data are shown in Table 1 and Table 2, respectively.

*3β,5β,11α,14β,19-Pentahydroxy-12-oxo-bufa-20,22-dienolide* (**5**): White powder (15.4 mg); ${\left[\alpha \right]}_{D}^{20}$ = +47.341° (*c* 0.54, MeOH); ECD (*c* 2.23 × 10^−4^ M, MeOH) λ_max_ (Δε) 217 (+1.09), 245 (−0.05), 284 (+0.85); IR υ_max_: 3413 (OH), 2929 and 2879 (CH), 1709 (C=O), 1632 (C=C), 1539, 1250, 1131, 1019 cm^−1^; UV (UPLC-PDA) λ_max_ (nm): 299; HR-ESI-TOF-MS, *m*/*z*: 447.2021 [M − H]^−^ (calc. for C_24_H_31_O_8_ = 447.2024); ESI-MS/MS (TOF), *m*/*z*: 493 [M − H]^−^, 447 [M − H − 46]^−^; ^13^C- and ^1^H-NMR data are shown in Table 1 and Table 3, respectively.

*19-(Acetyloxy)-3β,5β,11α,14β-tetrahydroxyl12-oxo-bufa-20,22-dienolide* (**6**): White powder (14.7 mg); ${\left[\alpha \right]}_{D}^{20}$ = +54.800° (*c* 0.43, MeOH); ECD (*c* 2.04 × 10^−4^ M, MeOH) λ_max_ (Δε) 218 (+1.01), 245 (−0.21), 279 (+0.98); IR υ_max_: 3423 (OH), 2941 and 2879 (CH), 1711 (C=O), 1634 (C=C), 1540, 1247, 1133, 1029 cm^−1^; UV (UPLC-PDA) λ_max_ (nm): 301; HR-ESI-TOF-MS, *m*/*z*: 489.2125 [M − H]^−^ (calc. for C_26_H_33_O_9_ = 489.2130); ESI-MS/MS (TOF), *m*/*z*: 535 [M − H]^−^, 489 [M − H − 46]^−^; 447 [M − H – 46 − 42], 429 [M − H – 46 − 60]^−^; ^13^C- and ^1^H-NMR data are shown in Table 1 and Table 3, respectively.

*19-(Acetyloxy)-1β,3β,5β,11α,14β-pentahydroxy-12-oxo-bufa-20,22-dienolide* (**7**): White powder (4.8 mg); ${\left[\alpha \right]}_{D}^{20}$ = +18.070° (*c* 0.27, MeOH); ECD (*c* 1.97 × 10^−4^ M, MeOH) λ_max_ (Δε) 213 (+1.18), 246 (−0.12), 285 (+0.75); IR υ_max_: 3429 (OH), 2944 and 2880 (CH), 1712 (C=O), 1633 (C=C), 1539, 1247, 1124, 1036 cm^−1^; UV (UPLC-PDA) λ_max_ (nm): 298; HR-ESI-TOF-MS, *m*/*z*: 505.2090 [M − H]^−^ (calc. for C_26_H_33_O_10_ = 505.2079); ESI-MS/MS (TOF), *m*/*z*: 551 [M − H]^−^, 505 [M − H − 46]^−^, 463 [M − H − 46 − 42], 445 [M − H – 46 − 60]^−^; ^13^C- and ^1^H-NMR data are shown in Table 1 and Table 3, respectively.

*1β-(Acetyloxy)-3β,5β,11α,14β,19-pentahydroxy-12-oxo-bufa-20,22-dienolide* (**8**): White powder (4.6 mg); ${\left[\alpha \right]}_{D}^{20}$ = +23.600° (*c* 0.13, MeOH); ECD (*c* 1.97 × 10^−4^ M, MeOH) λ_max_ (Δε) 222 (+1.33), 284 (+0.85); IR υ_max_: 3441 (OH), 2947 and 2880 (CH), 1712 (C=O), 1632 (C=C), 1539, 1247, 1126, 1033 cm^−1^; UV (UPLC-PDA) λ_max_ (nm): 298; HR-ESI-TOF-MS, *m/z*: 505.2088 [M − H]^−^ (calc. for C_26_H_33_O_10_ = 505.2079); ESI-MS/MS (TOF), *m/z*: 551 [M − H]^−^, 505 [M − H − 46]^−^, 463 [M − H – 46 − 42], 445 [M − H – 46 − 60]^−^; ^13^C- and ^1^H-NMR data are shown in Table 1 and Table 3, respectively.

### 3.4. Acid Hydrolysis of Compound **3** and Determination of the Absolute Configuration of the Sugar

Compound **3** (0.50 mg) was hydrolysed in 1 mL solution of 3 M HCl (dioxane-H_2_O, 1:2) at 90 °C for 2 h. After dioxane was removed from the hydrolysis reaction mixture under a stream of nitrogen, aglycones were extracted with EtOAc (3 × 1 mL) and dried. The aqueous layer containing sugar was neutralized using an Amberlite IRA-400 ion exchange resin in its hydroxide form, concentrated under a stream of nitrogen at 60 °C and afterwards used for the sugar determination. To determine the absolute configuration of monosaccharide of the isolated compound, a modified method of Tanaka *et al.* was used [24]. Sugars of the sample were dissolved in pyridine (200 µL), to which 0.5 mg of l-cysteine methyl ester hydrochloride was added. The mixture was kept at 60 °C for 1 h. 0.2 µL of *o*-tolyl isothiocyanate was added to the mixture, which was heated at 60 °C for 1 h. After cooling, the reaction mixture was directly analysed by UPLC-ESI-MS/MS. Chromatographic separation was carried out on an Acquity BEH C18 column (100 × 2.1 mm, 1.7 μm; Waters). Mass spectrometry analyses were performed in a positive ionization mode, using the SRM method. Details of the analysis can be found in the work of Pérez, *et al.* [25]. l-Rhamnose (Rha) was identified on the basis of the retention time (Rt) and *m*/*z* value of an authentic standard, derivatized in the same way as the sample (Rt of Rha, for both the sample and standard, was 11.62 min).

## 4. Conclusions

In conclusion, roots of *Kalanchoe deigremontiana* are a rich source of various bufadienolides. In the present study, eight bufadienolides which have not been reported previously in the scientific literature, were isolated from the aqueous extract and named: 1β,3β,5β,14β,19-pentahydroxybufa-20,22-dienolide (**1**), 19-(acetyloxy)-1β,3β,5β,14β-tetrahydroxybufa-20,22-dienolide (**2**), 3β-(*O*-α-l-rhamnopyranosyl)-5β,11α,14β,19-tetrahydroxybufa-20,22-dienolide (**3**), 19-(acetyloxy)-3β,5β,11α, 14β-tetrahydroxy-bufa-20,22-dienolide (**4**), 3β,5β,11α,19-pentahydroxy-12-oxo-bufa-20,22-dienolide (**5**), 19-(acetyloxy)-3β,5β,11α,14β-tetrahydroxy-12-oxo-bufa-20,22-dienolide (**6**), 19-(acetyloxy)-1β,3β,5β,11α,14β-pentahydroxy-12-oxo-bufa-20,22-dienolide (**7**), and 1β-(acetyloxy)-3β,5β,11α,14β, 19-pentahydroxy-12-oxo-bufa-20,22-dienolide (**8**), together with seven known compounds: 11*α*,19-dihydroxytelocinobufagin (**9**), bersaldegenin-1-acetate (**10**), daigredorigenin-3-acetate (**11**), bersaldegenin-1,3,5-orthoacetate (**12**), bryotoxin B (**13**), bryophyllin B (**14**), and bersaldegenin (**15**). Most of these compounds are acetylated in position 19 or 1, and only one contains a sugar moiety—the bufadienolide **2**. The discovery of these new compounds from *K. daigremontiana* provides more insight into the bufadienolides of the *Kalanchoe* genus.

## Figures and Tables

**Table 1:** ^13^C-NMR data for compounds **1**–**8** in CD_3_OD (δ_C_ in ppm).

Position	1	2	3	4	5	6	7	8
1	71.7	69.9	22.4	22.1	21.9	22.0	71.0	74.1
2	34.6	34.3	27.4	28.8	29.0	28.6	34.6	32.1
3	68.1	67.9	76.0	68.6	68.9	68.4	67.9	67.8
4	39.3	39.5	36.4	38.6	38.4	38.5	40.0	38.9
5	76.5	75.3	77.5	75.9	78.1	75.9	76.1	76.5
6	37.1	36.9	36.2	36.8	36.5	36.6	37.0	36.5
7	24.6	24.6	24.7	24.8	24.8	24.5	24.6	24.6
8	42.5	42.6	41.2	41.5	39.8	40.1	40.1	39.8
9	42.5	42.2	45.7	45.4	43.9	43.6	46.3	46.5
10	47.0	47.0	45.3	45.7	45.4	46.1	49.3	49.3
11	23.7	24.0	69.6	69.8	75.1	75.5	74.9	74.9
12	42.1	42.0	51.6	52.0	214.5	214.2	213.9	214.2
13	49.6	49.6	50.1	50.1	63.6	63.6	63.4	63.4
14	85.9	85.9	85.5	85.4	85.6	85.6	85.4	85.4
15	33.1	33.1	33.1	32.9	32.7	32.7	32.9	32.9
16	29.8	29.7	29.6	29.6	29.0	29.0	28.9	28.8
17	52.1	52.1	51.9	51.9	42.1	42.0	42.1	42.1
18	17.3	17.3	18.4	18.2	17.9	18.0	17.7	17.5
19	62.7	63.9	65.3	67.8	65.9	67.5	64.3	61.2
20	125.0	124.9	124.4	124.5	123.1	123.0	123.0	123.1
21	150.5	150.5	150.6	150.7	151.6	151.6	151.7	151.7
22	149.3	149.3	149.1	149.1	149.1	149.0	149.0	149.1
23	115.5	115.5	115.5	115.5	115.9	115.9	115.9	115.8
24	164.8	164.7	164.7	164.7	164.4	164.4	164.4	164.4
1-*CO*CH_3_								172.6
1-CO*CH*_3_								21.8
19-*CO*CH_3_		173.0		173.0		172.8	173.1	
19-CO*CH*_3_		21.3		21.2		21.2	21.2	
Rha								
1′			100.9					
2′			72.6					
3′			72.5					
4′			73.8					
5′			70.6					
6′			18.0					

**Table 2:** ^1^H-NMR data for compounds **1**–**4** in CD_3_OD (δ_H_ in ppm, *J* in Hz) ^a^.

Position	1	2	3	4
1α	4.42, dd (3.3, 3.3)	4.36, dd (3.3, 3.3)	2.41, br d (13.6)	2.50, ddd (13.8, 3.0, 3.0)
1β			2.03, ddd (13.6, 13.6, 2.5)	1.73, ddd (13.9, 13.9, 3.5)
2α	1.98, ddd (14.9, 3.5, 3.5)	2.00, ddd (15.1, 2.7, 2.7)	1.96, ddd (14.1, 2.6, 2.6)	1.89, dddd (13.8, 13.8, 3.0, 3.0)
2β	2.08, dddd (15.0, 2.5, 2.5, 2.5)	2.09, dd (15.2, 1.5)	1.80, br d (15.0)	1.63, dddd (14.5, 3.3, 3.0, 3.0)
3α	4.22, br s	4.26, br s	4.15, br s	4.14, dddd (3.4, 3.4, 3.3, 3.3)
4α	2.32, dd (15.1, 3.9)	2.36, dd (15.2, 3.9)	2.16, dd (15.0, 2.6)	2.18, dd (14.7, 3.1)
4β	1.62, ddd (14.5, 2.0, 2.0) ^b^	1.67, d (15.0)	1.57, br d (14.6)	1.47, ddd (14.8, 3.0, 3.0) ^b^
6α	1.43, m	1.45, m	1.45, br d (14.4)	1.49, m
6β	1.65, ddd (14.1, 13.8, 4.8)	1.67, ddd (13.9, 13.9, 4.6)	1.83	1.84, ddd (13.5, 13.5, 4.5)
7α	1.29, dddd (13.7, 13.7, 12.7, 3.3)	1.32, m	1.30, dddd (14.2, 14.2, 13.9, 4.4)	1.30, m
7β	1.97, m	2.01, m	2.02, m	2.00, m
8	1.80, ddd (12.0, 11.7, 4.1)	1.85, ddd (12.0, 12.0, 4.0)	1.86	1.98, ddd (12.4, 12.4, 4.1)
9	1.46, m	1.54	1.76, dd (11.2, 11.2)	1.77, dd (11.8, 10.3)
11α	1.51, m	1.53		
11β	1.58, t (12.0)	1.53	3.93, ddd (10.8, 10.8, 4.1)	3.88, ddd (11.1, 11.0, 4.6)
12α	1.35, m	1.38, dd (13.0, 13.0)	1.53, dd (13.0, 11.5)	1.53, dd (13.2, 11.6)
12β	1.50, m	1.50, d (13.0)	1.67, dd (13.4, 4.2)	1.68, dd (13.4, 4.4)
15α	1.69	1.71	1.73	1.73
15β	2.05, m	2.07, m	2.14, m	2.13, m
16α	2.18, ddd (11.7, 9.4, 9.4)	2.19, dddd (9.4, 9.1, 9.1, 3.6)	2.20, m	2.22, m
16β	1.72	1.73	1.76	1.75
17	2.54, dd (9.6, 6.1)	2.55, dd (9.7, 6.0)	2.62, dd (9.2, 6.5)	2.61, dd (9.3, 6.3)
18	0.74, s	0.71, s	0.76, s	0.74, s
19-a	4.40, d (11.8)	4.81, d (12.1)	4.13, d (11.2)	4.45, d (11.9)
19-b	4.06, d (11.6)	4.62, d (11.8)	3.84, d (11.3)	4.42, d (11.9)
21	7.42, dd (2.6, 1.1)	7.42, dd (2.6, 1.0)	7.45, dd (2.5, 0.9)	7.45, dd (2.6, 1.1)
22	7.98, dd (9.7, 2.6)	7.98, dd (9.7, 2.6)	7.94, dd (9.8, 2.5)	7.96, dd (9.7, 2.6)
23	6.28, dd (9.7, 1.1)	6.28, dd (9.8, 1.0)	6.28, dd (9.7, 0.8)	6.28, dd (9.7, 1.0)
19-CO*CH*_3_		2.05, s		2.07, s
Rha				
1′			4.85, d (1.6)	
2′			3.78, dd (3.3, 1.7)	
3′			3.63, dd (9.5, 3.5)	
4′			3.40, dd (9.5, 9.5)	
5′			3.65, dq (9.5, 6.2)	
6′			1.26, d (6.3)	

^a^ Overlapped signals were reported without designating multiplicity; ^b^ Long-range COSY between H-2β and H-4β.

**Table 3:** ^1^H-NMR data for compounds **5**–**8** in CD_3_OD (δ_H_ in ppm, *J* in Hz) ^a^.

Position	5	6	7	8
1α	2.43, ddd (14.2, 3.4, 3.4)	2.51, m	5.24, br s	6.52, dd (2.8, 2.8)
1β	2.07, ddd (14.1, 14.1, 3.8)	1.75, m		
2α	1.85, dddd (14.4, 14.4, 3.3, 3.3)	1.78, m	2.14, ddd (15.5, 3.4, 3.4)	2.11, m
2β	1.64, ddd (14.4, 3.2, 3.2)	1.62, ddd (13.7, 2.8, 2.8)	2.04, br d (15.5)	2.11, m
3α	4.08, dddd (2.8, 2.8, 2.8, 2.8)	4.11, br s	4.22, br s	4.22, dddd (3.3, 3.3, 3.3, 3.3)
4α	2.10, dd (15.0, 3.1)	2.11, dd (14.9, 2.8)	2.27, dd (15.2, 3.7)	2.25, dd (14.9, 3.5)
4β	1.45, ddd (14.8, 2.7, 2.7) ^b^	1.49, br d (13.7)	1.68, ddd (14.9, 2.2, 2.2) ^b^	1.61, dd (14.9, 2.8)
6α	1.51, ddd (13.3, 3.6, 3.6)	1.48, ddd (14.1, 3.7, 3.7)	1.49, ddd (13.2, 3.9, 3.9)	1.48, ddd (13.8, 3.8, 2.7)
6β	1.97, ddd (13.4, 13.4, 4.5)	1.85, m	1.77, ddd (13.6, 13.6, 4.6)	1.80, ddd (13.7, 13.6, 4.6)
7α	1.34, dddd (13.6, 13.5, 13.5, 4.3)	1.36, dddd (13.7, 13.7, 13.7, 4.7)	1.36, dddd (14.0, 14.0, 13.9, 4.4)	1.36, dddd (14.0, 14.0, 13.7, 4.3)
7β	2.10, dddd (13.6, 4.5, 4.4, 2.6)	2.11, m	2.13, dddd (13.5, 4.0, 4.0, 4.0)	2.13, m
8	2.42, ddd (12.4, 12.3, 4.2)	2.43, ddd (12.4, 12.4, 4.2)	2.38, ddd (12.3, 12.1, 4.0)	2.49, ddd (12.4, 12.4, 4.0)
9	1.80, dd (11.6, 11.6)	1.83, dd (11.6, 11.6)	1.67, dd (11.6, 11.6)	1.68, dd (11.4, 11.4)
11β	4.71, d (11.2)	4.61, d (11.2)	4.65, d (11.1)	5.07, d (10.9)
15α	1.32, m	1.34, m	1.29, m	1.28, m
15β	1.73	1.75	1.75	1.72
16α	2.00, dddd (10.0, 10.0, 9.6, 3.5)	2.02, dddd (9.8, 9.8, 9.3, 4.7)	2.01, dddd (9.6, 9.6, 9.5, 3.9)	2.00, m
16β	1.73	1.74	1.74	1.75
17	4.11, dd (9.9, 7.0)	4.12, dd (9.9, 7.0)	4.11, dd (9.7, 6.9)	4.11, dd (9.8, 6.8)
18	0.92, s	0.91, s	0.91, s	0.95, s
19-a	4.19, d (11.3)	4.60, d (12.0)	5.00, d (12.2)	4.37, d (11.5)
19-b	4.01, d (11.3)	4.50, d (12.0)	4.85, d (12.0)	4.29, d (11.6)
21	7.52, dd (2.5, 0.9)	7.53, dd (2.5, 0.8)	7.52, dd (2.6, 0.6)	7.52, br d (2.7)
22	7.91, dd (9.7, 2.6)	7.92, dd (9.7, 2.6)	7.92, dd (9.8, 2.6)	7.93, dd (9.7, 2.7)
23	6.31, dd (9.7, 1.0)	6.31, dd (9.7, 0.7)	6.31, dd (9.7, 0.8)	6.31, dd (9.7, 0.9)
1-CO*CH*_3_				2.04, s
19-CO*CH*_3_		2.08, s	2.08, s	

^a^ Overlapped signals were reported without designating multiplicity; ^b^ Long-range COSY between H-2β and H-4β.

## Supplementary Materials

Supplementary materials can be accessed at: http://www.mdpi.com/1420-3049/21/3/243/s1.

## References

[1] Herrera I., Nassar J.M. (2009). Reproductive and recruitment traits as indicators of the invasive potential of *Kalanchoe daigremontiana* (Crassulaceae) and *Stapelia gigantean* (Apocynaceae) in a neotropical arid zone. J. Arid Environ..

[2] Anisimov M.M., Gerasimenko E.L., Chaikina E.L., Serebryakov Y.M. (2009). Biological activity of metabolites of the herb *Kalanchoe daigremontiana* (Hamet de la Bathie) Jacobs et Perr. Biol. Bull..

[3] Misra S.B., Dixit S.N. (1979). Antifungal activity of leaf extracts of some higher plants. Acta Bot. Indica.

[4] Nassis C.Z., Haebisch E.M.A.B., Giesbrecht A.M. (1992). Antihistamine activity of *Bryophillum calycinum*. Braz. J. Med. Res..

[5] Moraes V.L.G., Santos L.F.M., Castro S.B., Loureiro L.H., Lima O.A., Souza M.L.M., Yien L.M.K., Rossi-Bergmann B., Costa S.S. (1994). Inhibition of lymphocyte activation by extracts and fractions of *Kalanchoe*, *Alternanthera*, *Paullinea*, and *Mikania* species. Phytomedicine.

[6] Rossi-Bergmann B., Costa S.S., Borges M.B.S., da Silva S.A., Noleto G.R., Souza M.L.M., Moraes V.L.G. (1994). Immunosuppressive effect of the aqueous extract of *Kalanchoe pinnata* in mice. Phytother. Res..

[7] Da-Silva S.A.G., Costa S.S., Mendonca S.C.F., Silva E.M., Moraes V.L.G., Rossi-Bergmann B. (1995). Therapeutic effect of oral *Kalanchoe pinnata* leaf extract in murine leishmaniasis. Acta Trop..

[8] Da-Silva S.A.G., Costa S.S., Rossi-Bergmann B. (1999). The anti-leishmanial effect of *Kalanchoe* is mediated by nitric oxide intermediates. Parasitology.

[9] Supratman U., Fujita T., Akiyama K., Hayashi H. (2000). New insecticidal bufadienolide, bryophyllin C, from *Kalanchoe pinnata*. Biosci. Biotechnol. Biochem..

[10] Supratman U., Fujita T., Akiyama K., Hayashi H. (2001). Insecticidal compounds from *Kalanchoe daigremontiana* × *tubiflora*. Phytochemistry.

[11] Supratman U., Fujita T., Akiyama K., Hayashi H., Murakami A., Sakai H., Koshimizu K., Ohigashi H. (2001). Anti-tumor promoting activity of bufadienolides from *Kalanchoe pinnata* and *K. daigremontiana* × *tubiflora*. Biosci. Biotechnol. Biochem..

[12] Yadav N.P., Dixit V.K. (2003). Hepatoprotective activity of leaves of *Kalanchoe pinnata* Pers. J. Ethnopharmacol..

[13] Costa S.S., de Lourdes M., de Souza M.L.M., Ibrahim T., Oliveira de Melo G.M., de Almeida A.P., Guette C., Férézou J.P., Koatz V.L.G. (2006). Kalanchosine dimalate, an anti-inflammatory salt from *Kalanchoe brasiliensis*. J. Nat. Prod..

[14] Lai Z.R., Ho Y.L., Huang S.C., Huang T.H., Lai S.C., Tsai J.C., Wang C.Y., Huang G.J., Chang Y.S. (2011). Antioxidant, anti-inflammatory and antiproliferative activities of *Kalanchoe gracilis* (L.) dc stem. Am. J. Chin. Med..

[15] Cruz E.A., Reuter S., Martin H., Dehzad N., Muzitano M.F., Costa S.S., Rossi-Bergmann B., Buhl R., Stassen M., Taube C. (2012). *Kalanchoe pinnata* inhibits mast cell activation and prevents allergic airway disease. Phytomedicine.

[16] Krenn L., Kopp B. (1998). Bufadienolides from animal and plant sources. Phytochemistry.

[17] Tian H.-Y., Wang L., Zhang X.-Q., Zhang D.-M., Wang Y., Liu J.-S., Jiang R.-W., Ye W.-C. (2010). New bufadienolides and C_23_ steroids from the venom of *Bufo bufo gargarizans*. Steroids.

[18] Li X., Liu Y., Shen A., Wang C., Yan J., Zhao W., Liang X. (2014). Efficient purification of active bufadienolides by a class separation method based on hydrophilic solid-phase extraction and reversed-phase high performance liquid chromatography. J. Pharm. Biomed. Anal..

[19] Green B., Snatzke F., Snatzke G., Pettit G.R., Kamano Y., Niven M.L. (1985). Circular Dichroism of Bufadienolides. Croat. Chem. Acta.

[20] Goetz M.A., Wiemer D.F., Haynes L.W., Meinwald J., Eisner T. (1979). Lucibufagins. Part III. 11-Oxo-and 12-oxo-bufalins, defensive steroids from the fireflies *Photinus ignitus* and *P. marginellus (Coleoptera: Lampyrida)*. Helv. Chim. Acta.

[21] González A., Schroeder F.C., Attygale A.B., Svatoš A., Meinwald J., Eisner T. (1999). Metabolic transformations of acquired lucibufagins by firefly “femmes fatales”. Chemoecology.

[22] Wagner H., Lotter H., Fischer M. (1986). The toxic and sedative bufadienolides of *Kalanchoe daigremontiana* Hamet et Perr. Helv. Chim. Acta.

[23] Żuchowski J., Pecio Ł., Stochmal A. (2014). Novel Flavonol Glycosides from the Aerial Parts of Lentil (*Lens culinaris*). Molecules.

[24] Tanaka T., Nakashima T., Ueda T., Tomii K., Kouno I. (2007). Facile discrimination of aldose enantiomers by reversed-phase HPLC. Chem. Pharm. Bull..

[25] Pérez A.J., Simonet A.M., Calle J.M., Pecio Ł., Guerra J.O., Stochmal A., Macías F.A. (2014). Phytotoxic steroidal saponins from *Agave offoyana* leaves. Phytochemistry.

