# Supplementary material for: New Bufadienolides Isolated from the Roots of Kalanchoe daigremontiana (Crassulaceae)
Source: Molecules. 2016 Feb 24;21(3):243. doi: 10.3390/molecules21030243 (PMC6272856; doi:10.3390/molecules21030243)
Supplement: Supplementary file 1 [file molecules-21-00243-s001.pdf]

Supplementary Materials: New Bufadienolides Isolated from the Roots of *Kalanchoe daigremontiana* (Crassulaceae)

Barbara Moniuszko-Szajwaj, Łukasz Pecio, Mariusz Kowalczyk and Anna Stochmal

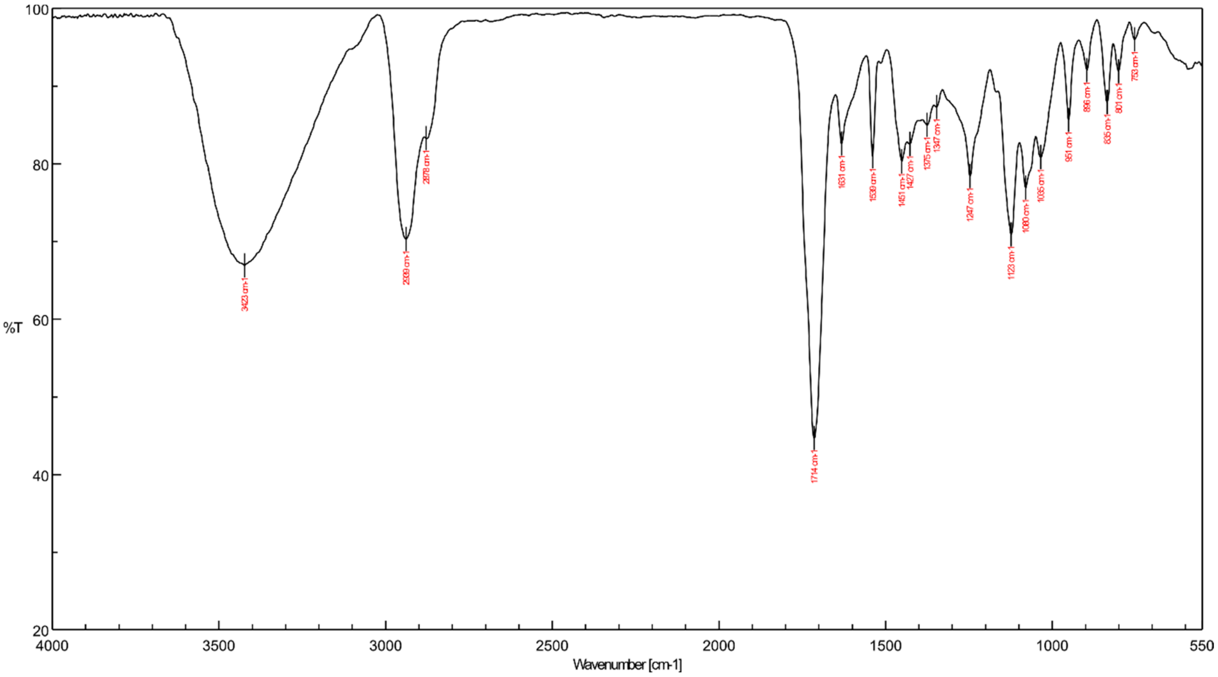

Figure S1. IR spectrum of compound **1**.

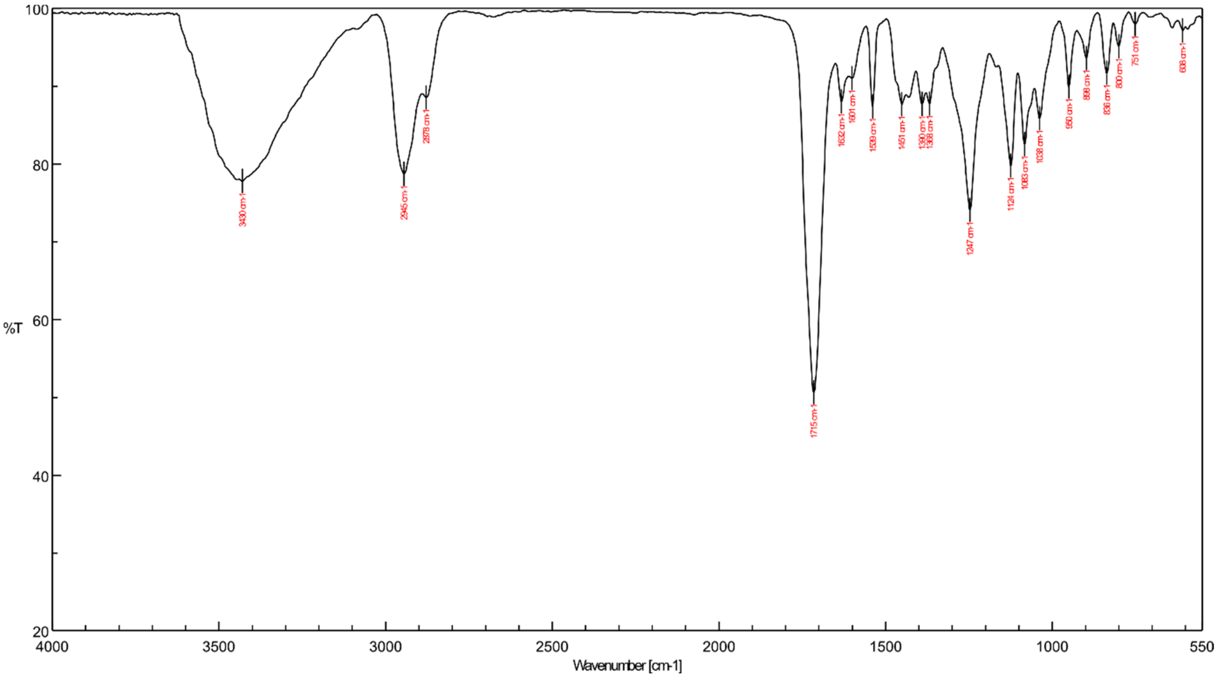

Figure S2. IR spectrum of compound **2**.

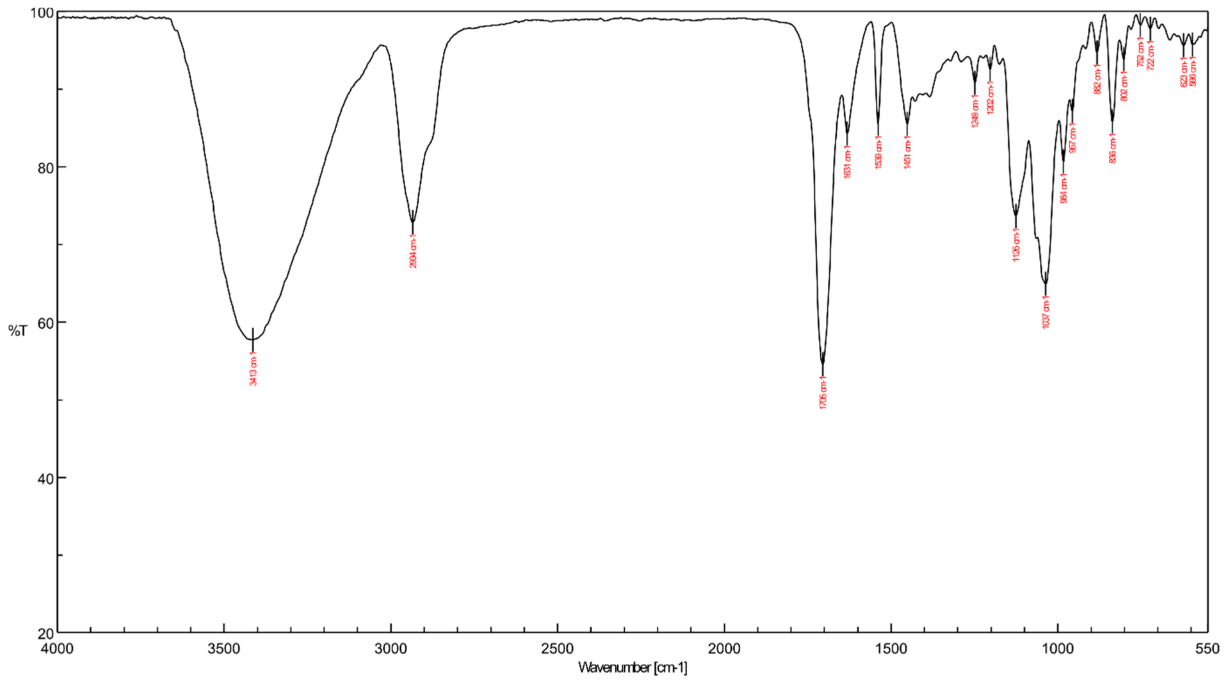

Figure S3. IR spectrum of compound **3**.

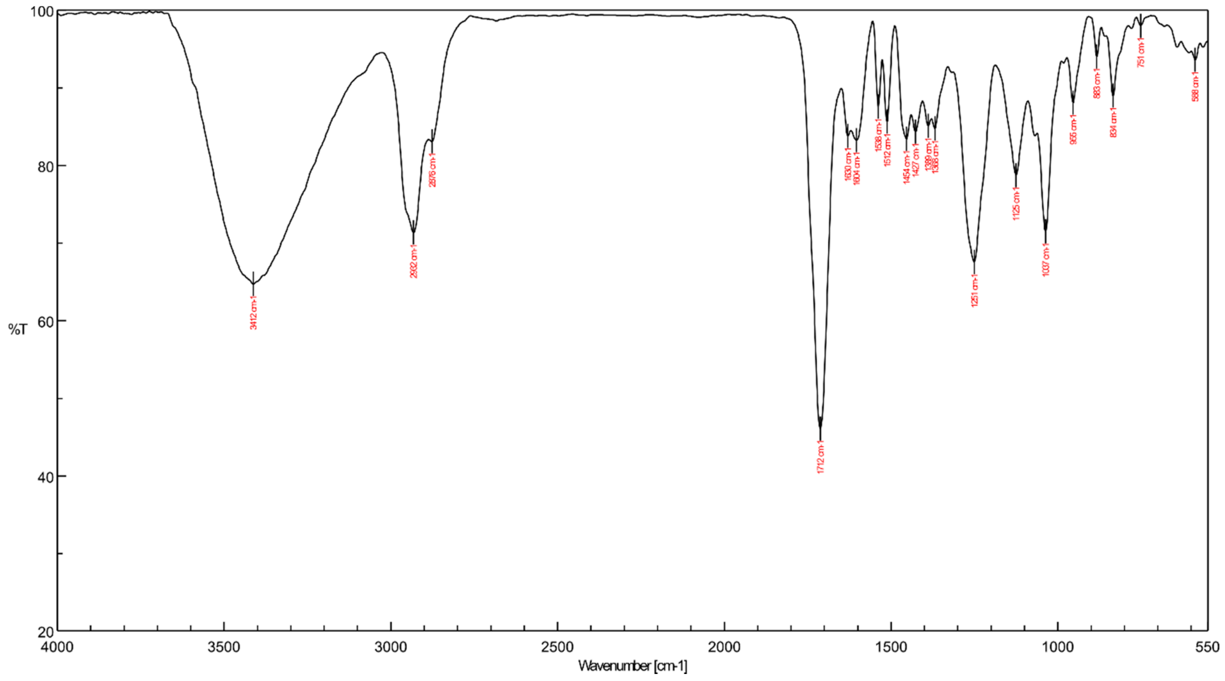

Figure S4. IR spectrum of compound **4**.

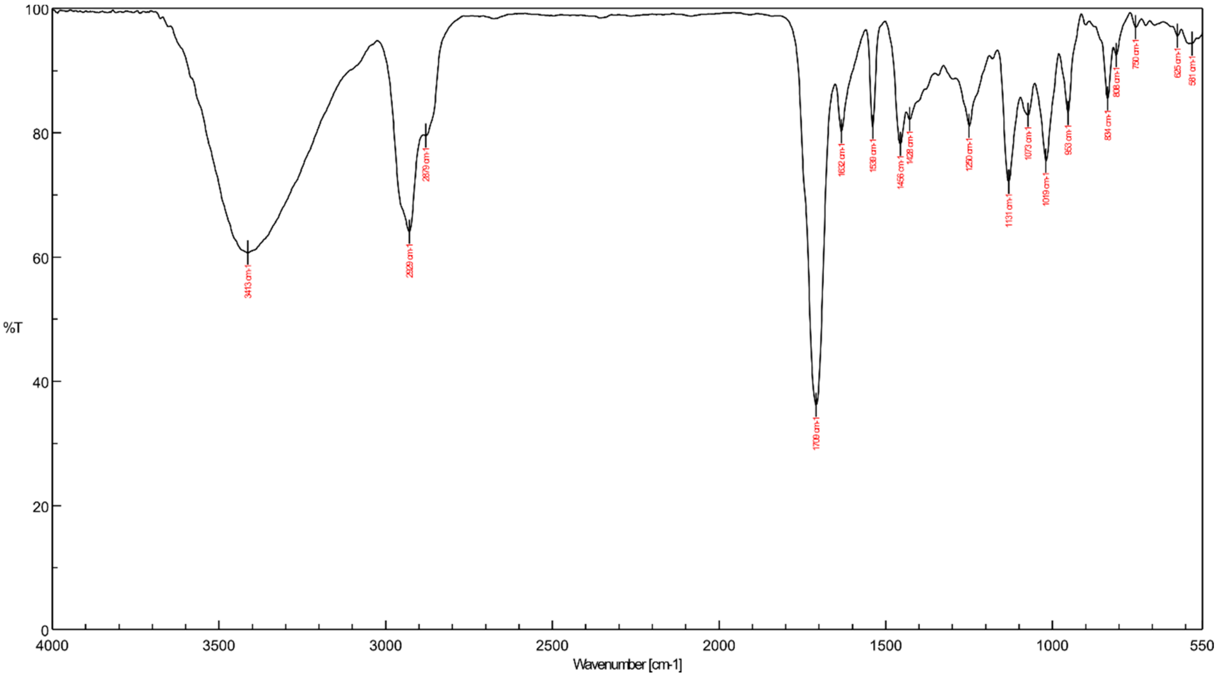

Figure S5. IR spectrum of compound **5**.

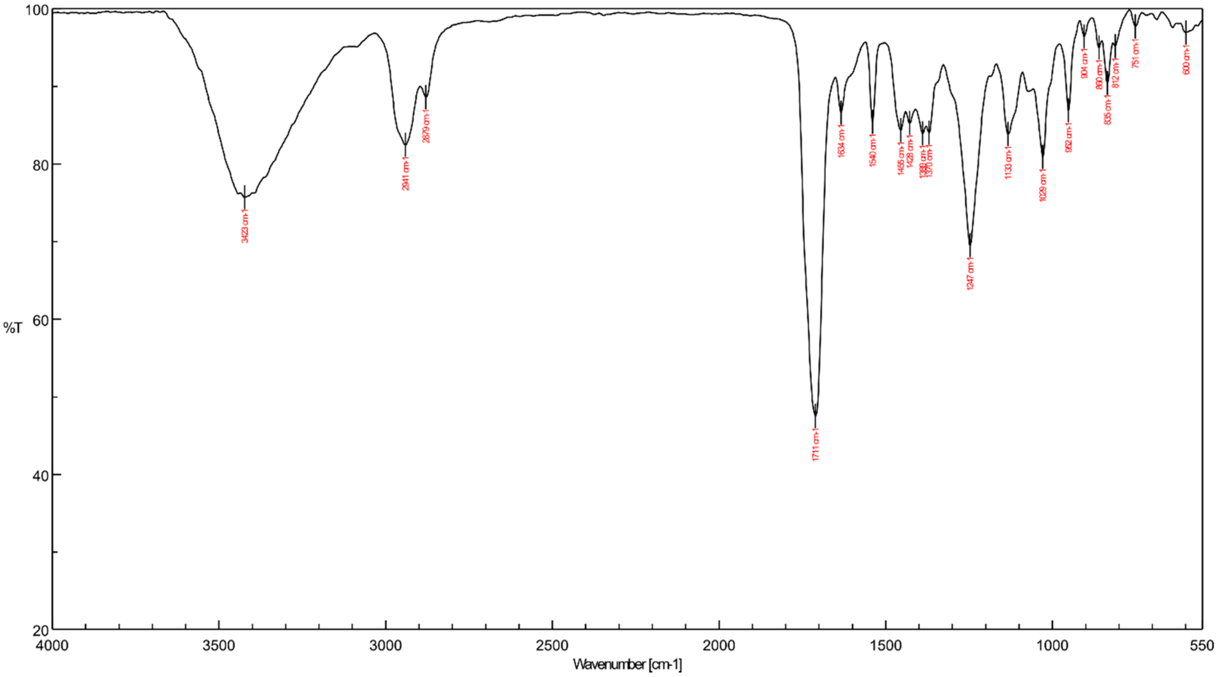

Figure S6. IR spectrum of compound **6**.

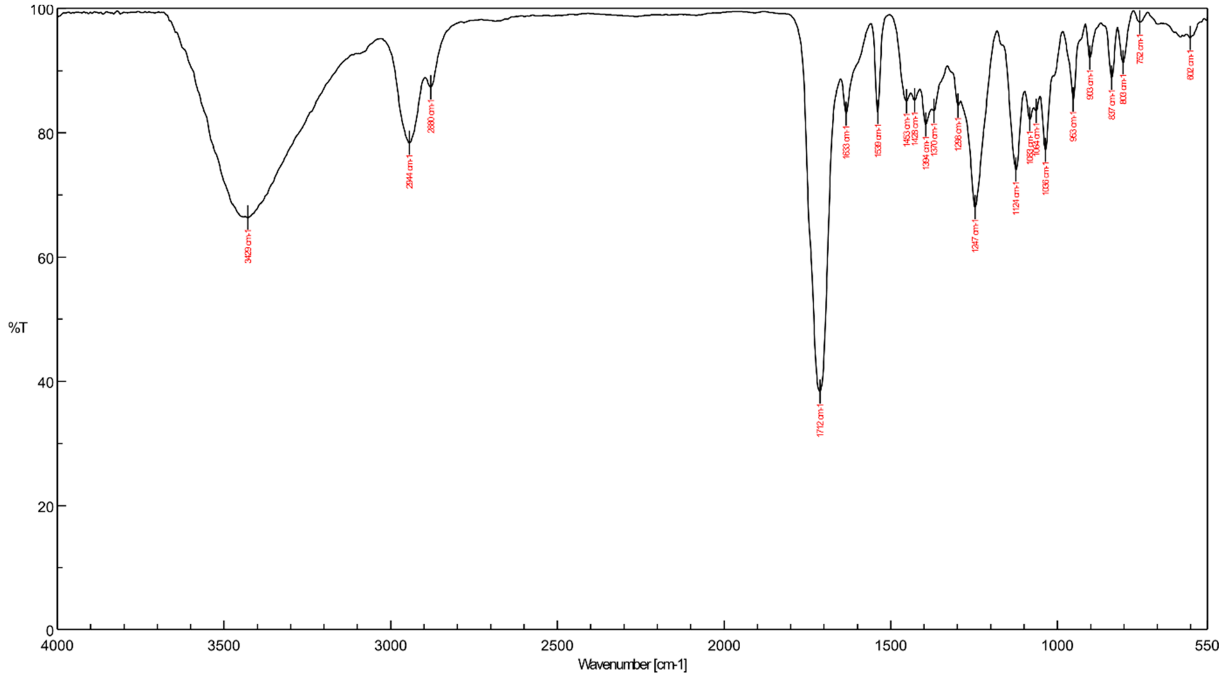

Figure S7. IR spectrum of compound **7**.

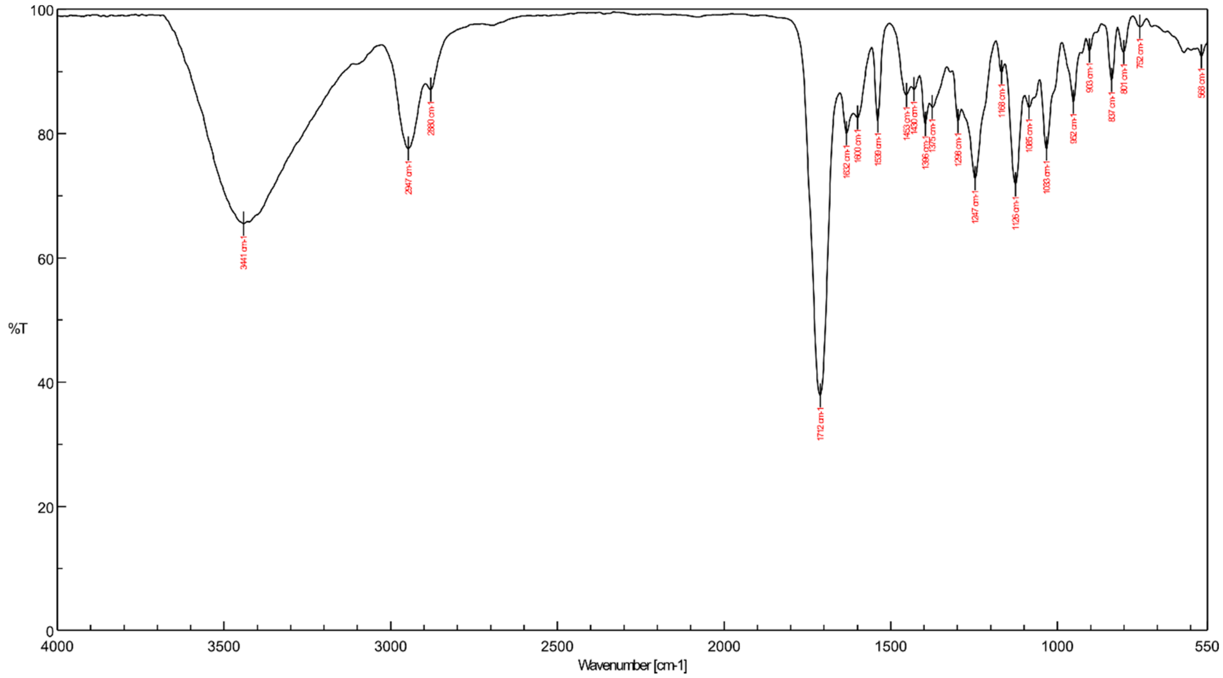

Figure S8. IR spectrum of compound **8**.

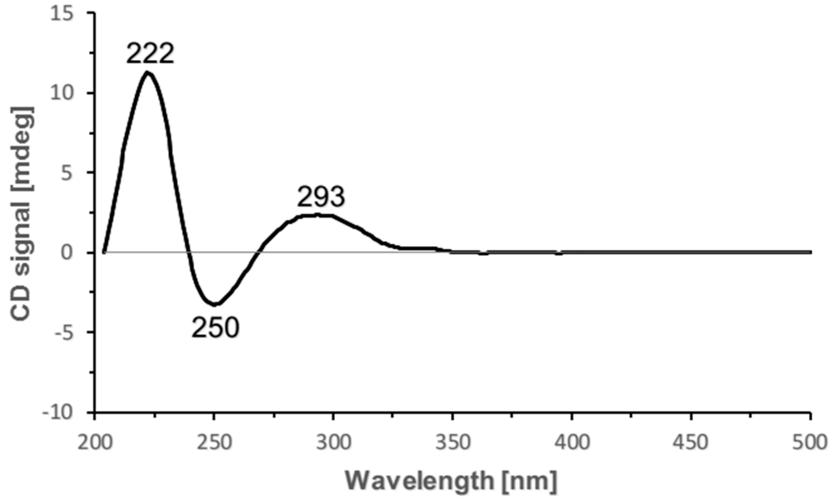

Figure S9. Experimental ECD spectrum of **1** (*c* 2.30 × 10^{-4} M, MeOH).

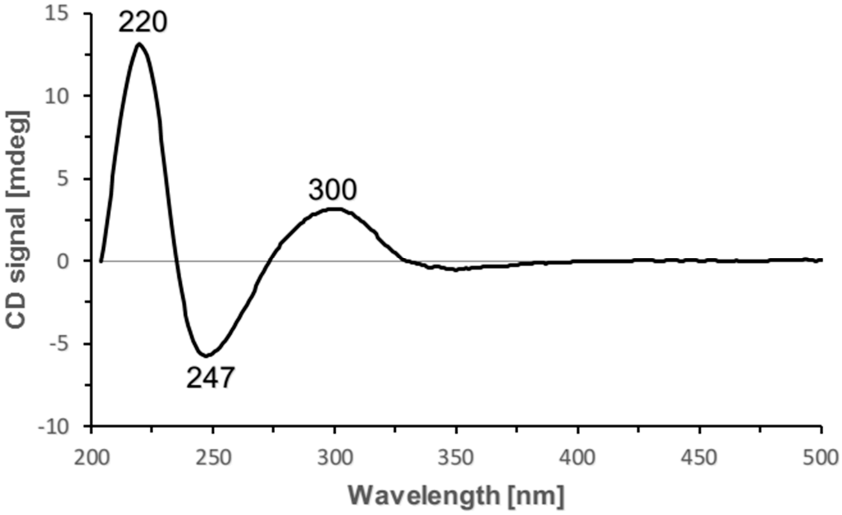

Figure S10. Experimental ECD spectrum of **2** (*c* 2.10 × 10^{-4} M, MeOH).

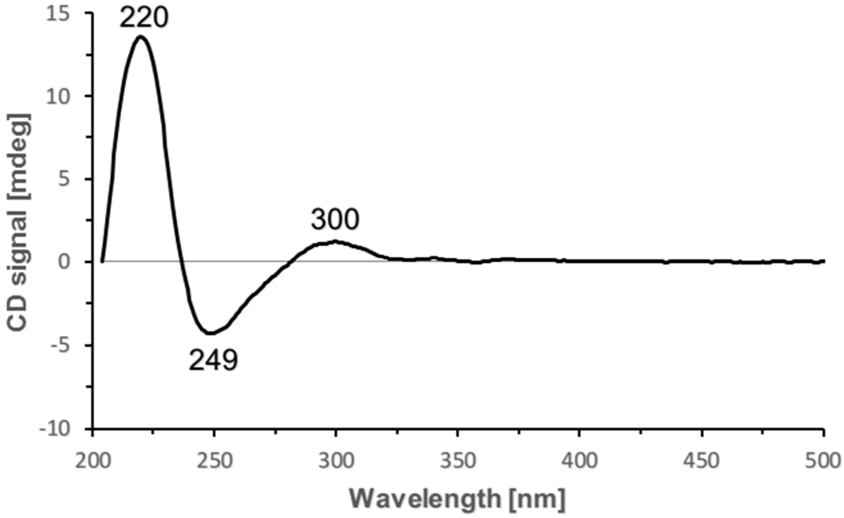

Figure S11. Experimental ECD spectrum of **3** (*c* 1.72 × 10^{-4} M, MeOH).

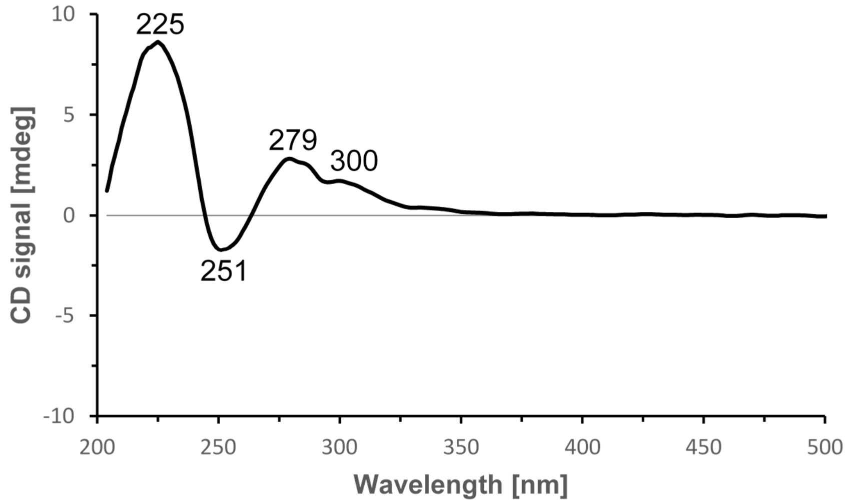

Figure S12. Experimental ECD spectrum of **4** (*c* 2.10 × 10^{-4} M, MeOH).

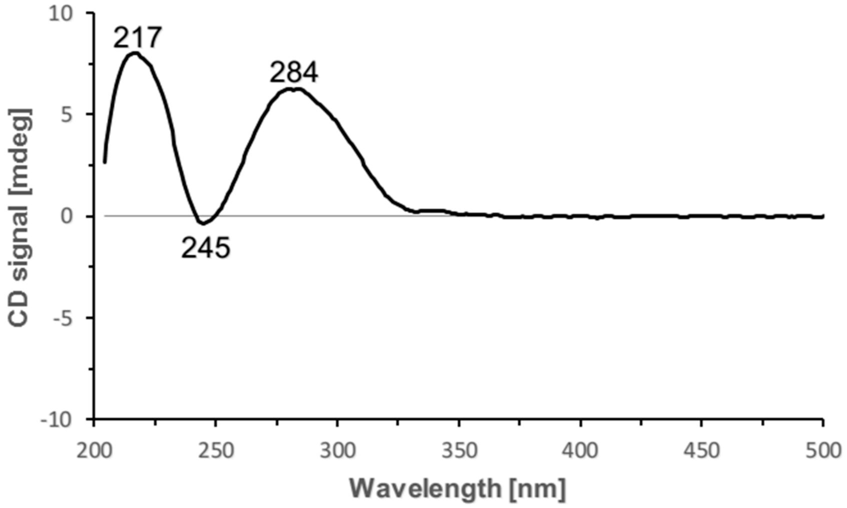

Figure S13. Experimental ECD spectrum of **5** (*c* 2.23 × 10^{-4} M, MeOH).

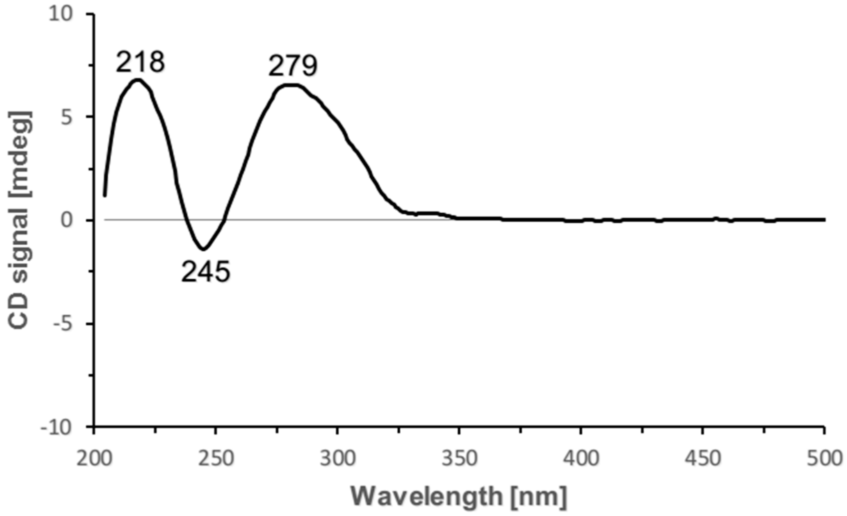

Figure S14. Experimental ECD spectrum of **6** (*c* 2.04 × 10^{-4} M, MeOH).

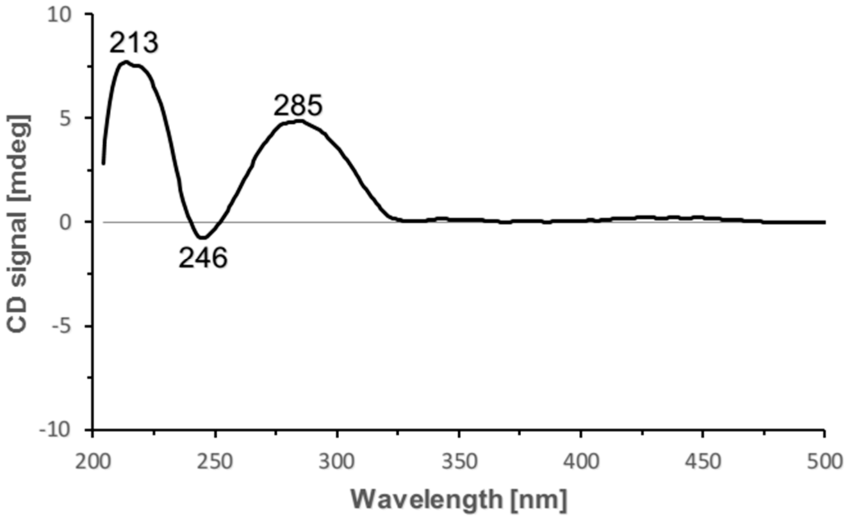

Figure S15. Experimental ECD spectrum of **7** (*c* 1.97 × 10^{-4} M, MeOH).

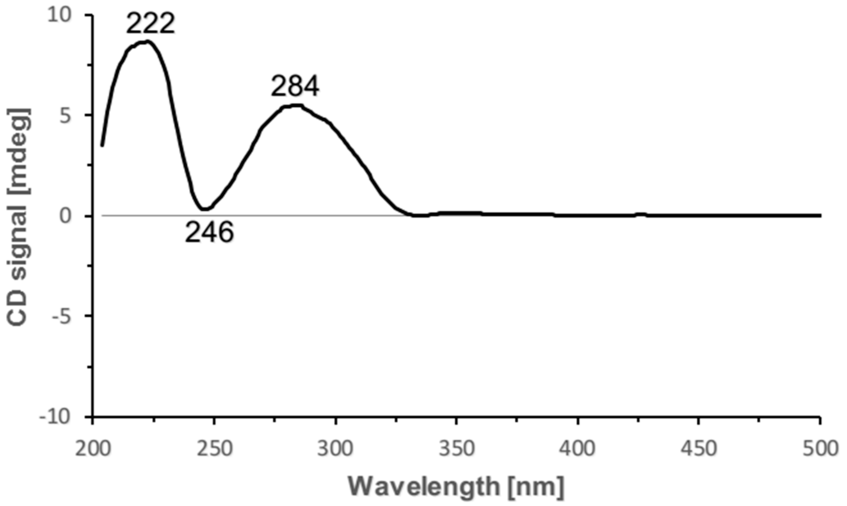

Figure S16. Experimental ECD spectrum of **8** (*c* 1.97 × 10^{-4} M, MeOH).

Table S1. ^{1}H- and ^{13}C-NMR data of compound **1** in DMSO-d_6.

Position	δ_H in ppm, J in Hz [a]	δ_C in ppm
1α	4.21, br s	68.9
2	1.86, m	33.6
3α	4.05, br s	65.7
4α	2.13, dd (14.6, 4.0)	38.4
4β	1.45, br d (14.3)	
5	-	73.9
6α	1.22, br d (13.2)	35.8
6β	1.48, ddd (13.7, 13.5, 4.6)	
7α	1.86, m	23.2
7β	1.09, dddd (13.0, 13.0, 12.8, 3.3)	
8	1.71, ddd (117.7, 11.5, 3.6)	40.6
9	1.31, m	40.5
10	-	45.6
11α	1.35	22.4
11β	1.56, m	
12α	1.20, m	40.4
12β	1.35, br d (11.5)	
13	-	47.9
14	4.16 (s, OH)	83.5
15α	1.55	31.8
15β	1.93, m	
16α	2.03, m	28.4
16β	1.57	
17	2.43, dd (9.6, 5.8)	50.0
18	0.62, s	16.7
19-a	4.16, d (10.9)	60.0
19-b	3.88, d (11.0)	
20	-	122.7
21	7.51, dd (2.6, 1.1)	149.2
22	7.92, dd (9.7, 2.6)	147.4
23	6.28, dd (9.7, 1.0)	114.2
24	-	161.3

[a] Overlapped signals were reported without designating multiplicity.

Table S2. ^{1}H- and ^{13}C-NMR data of compound **9** in CD_3OD.

11α,19-Dihydroxytelocinobufagin

$C_{24}H_{34}O_7$, M = 434.53 g/mol, HR-ESI-TOF-MS, *m*/*z*: 433.2230 $[M - H]^-$

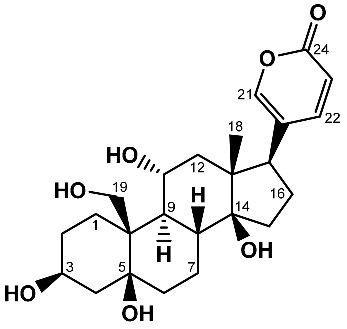

	δ_H in ppm, *J* in Hz [a]	δ_C in ppm
1α	2.36, br d (14.2)	21.8
1β	2.23, ddd (14.3, 14.3, 3.3)	
2α	1.68, ddd (14.3, 3.1, 3.1)	29.2
2β	1.94, dddd (13.5, 13.5, 3.5, 3.0)	
3α	4.11, br s	69.0
4α	2.16, dd (14.7, 3.1)	38.4
4β	1.44, ddd (15.0, 3.0, 3.0)	
5	-	78.7
6α	1.49, m	36.8
6β	1.84, ddd (13.5, 13.5, 4.5)	
7α	1.30, m	24.8
7β	2.00, m	
8	1.80	41.2
9	1.78	45.5
10	-	44.8
11β	3.82, ddd (10.5, 10.5, 4.0)	69.4
12α	1.52, dd (13.2, 11.5)	51.8
12β	1.66, dd (13.3, 4.2)	
13	-	50.0
14	-	85.4
15α	1.73	33.0
15β	2.11, m	
16α	2.20, m	29.6
16β	1.75	
17	2.61, dd (9.4, 6.2)	51.8
18	0.75, s	18.4
19-a	4.23, d (11.0)	65.9
19-b	3.81, d (11.1)	
20	-	124.4
21	7.44, dd (2.5, 1.1)	150.6
22	7.94, dd (9.7, 2.6)	149.1
23	6.28, dd (9.7, 1.0)	115.5
24	-	164.7

[a] Overlapped signals were reported without designating multiplicity.

Table S3. ^{1}H- and ^{13}C-NMR data of compound **9** in DMSO-d_6.

11α,19-Dihydroxytelocinobufagin

$C_{24}H_{34}O_7$, M = 434.53 g/mol, HR-ESI-TOF-MS, *m/z*: 433.2230, $[M - H]^-$

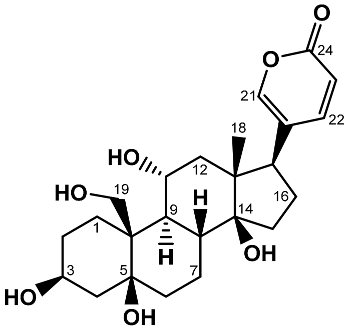

Position	δ_H in ppm, *J* in Hz [a]	δ_C in ppm
1α	2.26, br d (13.6)	20.5
1β	1.94, ddd (13.6, 13.5, 3.1)	
2α	1.47, br d (13.9)	28.1
2β	1.81, dddd (14.5, 14.5, 3.5, 3.5)	
3α	3.96, br s	66.5
3β	5.24, d (4.8, OH)	
4α	2.01, dd (14.5, 3.1)	37.4
4β	1.25, ddd (14.5, 2.6, 2.6)	
5	5.64, s (OH)	76.2
6α	1.30, m	35.8
6β	1.70	
7α	1.10, dddd (12.9, 12.9, 12.9, 3.5)	23.4
7β	1.89, m	
8	1.72	39.4
9	1.59, dd (12.0, 10.5)	43.8
10	-	43.3
11α	4.15, d (5.5, OH)	67.3
11β	3.71, dddd (10.0, 10.0, 5.3, 4.5)	
12α	1.35, dd (13.4, 10.8)	50.5
12β	1.51, dd (13.3, 4.1)	
13	-	48.4
14	4.15, s (OH)	83.2
15α	1.59	31.8
15β	1.98, m	
16α	2.04, m	28.2
16β	1.60	
17	2.50	49.7
18	0.61, s	17.9
19-a	3.95, dd (11.1, 3.3)	63.9
19-b	3.66, dd (10.9, 5.9)	
19	4.82, dd (6.0, 3.4, OH)	
20	-	122.3
21	7.54, dd (2.6, 1.1)	149.3
22	7.86, dd (9.8, 2.6)	147.2
23	6.28, dd (9.7, 1.0)	114.2
24	-	161.3

[a] Overlapped signals were reported without designating multiplicity.

Table S4. ^{1}H- and ^{13}C-NMR data of compound **10** in CD_3OD.

**Bersaldegenin 1-Acetate**
$C_{26}H_{34}O_8$, M = 474.55 g/mol, HR-ESI-TOF-MS, *m*/*z*: 473.2184 $[M-H]^-$

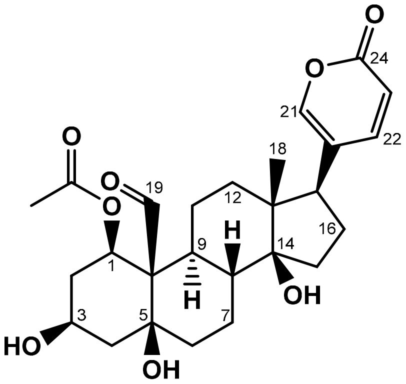

	δ_H **in ppm, *J* in Hz** [a]	δ_C **in ppm**
1α	5.70, dd (2.8, 2.8)	72.2
2α	1.94, ddd (15.7, 3.1, 3.1)	31.7
2β	2.12, dddd (15.9, 2.7, 2.6, 2.6)	
3α	4.23, dddd (3.0, 3.0, 3.0, 3.0)	67.2
4α	2.34, dd (15.0, 2.9)	38.7
4β	1.71, ddd (14.9, 2.8, 2.8)	
5	-	75.1
6α	1.76, m	37.2
6β	2.32, m	
7α	1.39	25.2
7β	2.17, m	
8	1.54	43.8
9	1.64	43.3
10	-	58.3
11α	1.62	23.0
11β	1.39	
12α	1.40	40.8
12β	1.51	
13	-	49.3
14	-	85.3
15α	1.66, dd (12.8, 7.0)	32.6
15β	2.05, ddd (12.8, 9.8, 9.8)	
16α	2.18, ddd (12.7, 9.3, 9.3)	29.5
16β	1.73, dddd (12.8, 9.7, 7.0, 6.5)	
17	2.54, dd (9.8, 6.6)	51.9
18	0.63, s	16.9
19	10.05, s	207.2
20	-	124.8
21	7.42, dd (2.6, 1.1)	150.6
22	7.96, dd (9.7, 2.6)	149.2
23	6.27, dd (9.7, 1.0)	115.5
24	-	164.7
1-<u>CO</u>CH_3	-	172.0
1-CO<u>CH_3</u>	1.94, s	21.3

[a] Overlapped signals were reported without designating multiplicity.

Table S5. ^{1}H- and ^{13}C-NMR data of compound **11** in CD_3OD.

Daigredorigenin 3-Acetate

$C_{26}H_{36}O_7$, M = 460.57 g/mol, HR-ESI-TOF-MS, *m*/*z*: 459.2388 [M − H]⁻

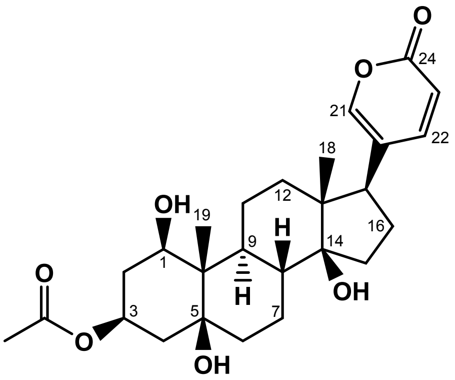

	δ_H in ppm, *J* in Hz [a]	δ_C in ppm
1α	3.89, dd (3.5, 3.5)	74.0
2	2.06, dd (3.8, 3.8)	31.9
3α	5.20, dddd (4.3, 4.3, 4.3, 4.3)	70.9
4α	2.43, dd (15.8, 4.6)	36.5
4β	1.71, d (15.8)	
5	-	75.3
6α	1.35, m	36.0
6β	1.75, ddd (13.8, 13.8, 4.9)	
7α	1.26, dddd (13.1, 13.1, 13.0, 3.8)	24.8
7β	1.98, m	
8	1.72, ddd (12.0, 12.0, 4.0)	42.0
9	1.48, ddd (11.5, 11.5, 3.9)	41.9
10	-	43.9
11α	1.34	22.7
11β	1.34	
12α	1.43, ddd (13.4, 13.4, 3.8)	41.5
12β	1.50, d (13.0)	
13	-	49.6
14	-	85.7
15α	1.71	33.1
15β	2.08, m	
16α	2.19, m	29.7
16β	1.73	
17	2.55, dd (9.6, 6.0)	52.1
18	0.72, s	17.2
19	1.21, s	13.2
20	-	124.9
21	7.43, dd (2.7, 1.1)	150.5
22	7.98, dd (9.7, 2.5)	149.3
23	6.28, dd (9.7, 1.0)	115.5
24	-	164.8
3-<u>CO</u>CH3	-	172.6
3-CO<u>CH3</u>	2.01, s	21.6

[a] Overlapped signals were reported without designating multiplicity.

Table S6. ^{1}H- and ^{13}C-NMR data of compound **12** in CD_3OD.

Bersaldegenin 1,3,5-orthoacetate

$C_{26}H_{32}O_7$, M = 456.54 g/mol, HR-ESI-TOF-MS, *m/z*: 455.2077 [M − H]⁻

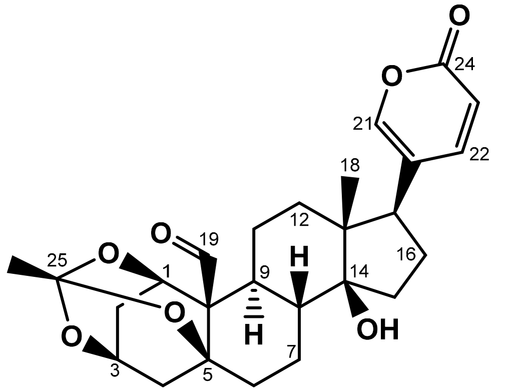

	δH in ppm, *J* in Hz [a]	δC in ppm
1α	4.59, dd (3.7, 1.8)	72.4
2α	1.86, ddd (13.6, 1.7, 1.7)	28.1
2β	2.36, dddd (13.6, 3.5, 3.5, 3.5)	
3α	4.28, dddd (4.0, 2.0, 2.0, 2.0)	68.7
4α	2.40, dd (14.0, 1.5)	34.5
4β	1.88, ddd (13.6, 3.1, 3.1)	
5	-	76.1
6α	1.59, ddd (13.2, 4.4, 2.8)	33.8
6β	2.39, ddd (13.6, 13.5, 4.8)	
7α	1.46, m	23.4
7β	2.13, dddd (11.3, 4.9, 3.2, 3.2)	
8	1.63, ddd (12.1, 12.0, 3.5)	43.3
9	1.71, ddd (13.5, 12.6, 4.0)	41.9
10	-	54.4
11α	1.48, m	21.8
11β	1.40	
12α	1.40	41.0
12β	1.49, ddd (13.2, 3.5, 3.5)	
13	-	49.8
14	-	85.0
15α	1.67, dd (12.1, 8.3)	32.5
15β	2.07, ddd (13.0, 9.9, 9.9)	
16α	2.18, ddd (12.2, 9.4, 9.4)	29.6
16β	1.73, dddd (12.2, 9.8, 8.0, 6.6)	
17	2.54, dd (9.7, 6.5)	52.0
18	0.64, s	17.0
19	10.1, d (1.3)	208.2
20	-	124.8
21	7.42, dd (2.6, 1.1)	150.6
22	7.96, dd (9.7, 2.6)	149.2
23	6.27, dd (9.7, 1.0)	115.5
24	-	164.7
25	-	112.0
26	1.29, s	26.1

[a] Overlapped signals were reported without designating multiplicity.

Table S7. ^{1}H- and ^{13}C-NMR data of compound **13** in CD_3OD.

Bryotoxin B

$C_{26}H_{32}O_9$, M = 488.53 g/mol, HR-ESI-TOF-MS, *m/z*: 487.1976 $[M - H]^-$

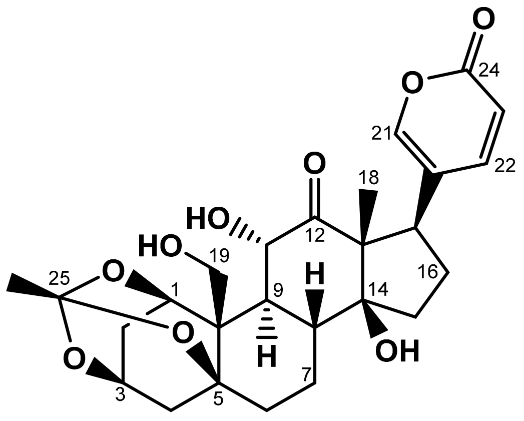

	δH in ppm, *J* in Hz[a]	δC in ppm
1α	5.09, ddd (4.4, 1.4, 1.4)	72.2
2α	2.04, ddd (13.8, 1.6, 1.6)	28.6
2β	2.32, ddd (13.9, 6.3, 3.4)	
3α	4.25, dddd (6.0, 3.8, 2.1, 2.1)	69.5
4α	2.30, dd (13.6, 1.9)	34.8
4β	1.79, ddd (13.5, 3.8, 2.3)	
5	-	76.6
6α	1.32, ddd (13.0, 4.0, 2.5)	33.2
6β	1.65, ddd (13.3, 13.2, 4.6)	
7α	1.45, dddd (13.8, 13.8, 13.8, 4.4)	22.6
7β	2.08, dddd (13.7, 4.5, 4.4, 4.4)	
8	2.53, ddd (12.4, 12.4, 4.5)	39.8
9	1.77, dd (11.8, 11.8)	45.0
10	-	45.4
11β	4.93, d (11.4)	75.2
12	-	213.8
13	-	63.9
14	-	85.6
15α	1.36, m	32.9
15β	1.78	
16α	2.01, m	29.1
16β	1.75	
17	4.13, dd (9.7, 6.9)	41.8
18	0.95, s	17.9
19-a	4.46, dd (11.8, 1.1)	60.6
19-b	4.30, d (11.8)	
20	-	123.1
21	7.52, dd (2.7, 1.0)	151.6
22	7.92, dd (9.7, 2.6)	149.1
23	6.31, dd (9.7, 1.1)	115.9
24	-	164.4
25	-	111.2
26	1.35, s	26.3

[a] Overlapped signals were reported without designating multiplicity.

Table S8. ^{1}H- and ^{13}C-NMR data of compound **14** in CD_3OD.

**Bryophyllin B**
$C_{26}H_{34}O_9$, M = 490.55 g/mol, HR-ESI-TOF-MS, *m*/*z*: 489.2133 [M − H]⁻

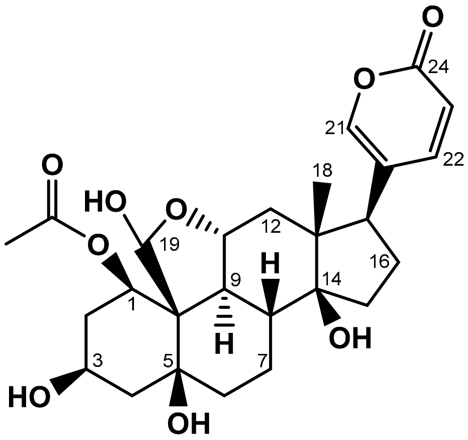

	δ_H in ppm, *J* in Hz [a]	δ_C in ppm
1α	4.69, dd (12.5, 3.9)	73.5
2α	1.55, ddd (12.1, 12.1, 12.1)	39.3
2β	2.11, m	
3α	3.79, dddd (10.8, 10.8, 4.9, 4.9)	65.7
4α	1.87, ddd (11.6, 11.6)	47.1
4β	1.93, dd (13.1, 5.0)	
5	-	76.9
6α	1.97	35.2
6β	1.58, ddd (15.6, 9.9, 9.9)	
7α	1.22, dddd (13.2, 13.2, 9.1, 9.1)	20.6
7β	1.96	
8	2.46, ddd (13.1, 13.1, 3.1)	40.0
9	1.30, dd (11.4, 11.4)	49.7
10	-	54.7
11β	3.99, ddd (11.2, 11.2, 3.4)	80.5
12α	1.46, dd (11.9, 11.9)	48.0
12β	2.03, dd (11.8, 3.4)	
13	-	53.2
14	-	86.6
15α	1.57, dd (13.5, 7.9)	32.2
15β	1.94, m	
16α	2.21, ddd (11.6, 8.5, 8.5)	31.3
16β	1.84, m	
17	2.71, dd (9.3, 7.4)	51.8
18	0.85, s	20.5
19	5.76, s	105.1
20	-	124.1
21	7.45, dd (2.6, 1.1)	150.6
22	7.87, dd (9.7, 2.6)	149.0
23	6.29, dd (9.7, 1.0)	115.6
24	-	164.6
1-$\underline{CO}CH_3$	-	172.4
1-$CO\underline{CH_3}$	2.08, s	21.0

[a] Overlapped signals were reported without designating multiplicity.

Table S9. ^{1}H- and ^{13}C-NMR data of compound **15** in CD_3OD.

Bersaldegenin

$C_{24}H_{32}O_7$, M = 432.51 g/mol, HR-ESI-TOF-MS, *m*/*z*: 431.2074 [M − H]$^-$

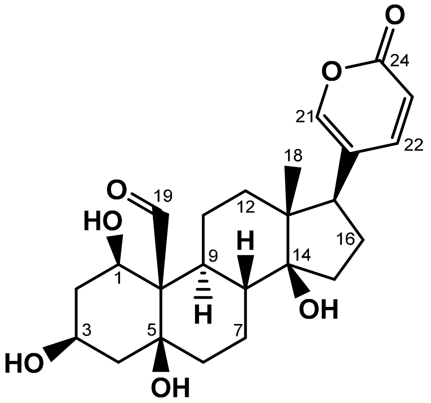

	**δ$_H$ in ppm, *J* in Hz** [a]	**δ$_C$ in ppm**
1α	4.65, dd (3.1, 3.1)	70.7
2α	1.95, ddd (14.9, 3.2, 3.2)	34.1
2β	2.11, ddd (14.9, 4.9, 3.2)	
3α	4.23, br s	67.6
4α	2.31, dd (15.2, 4.2)	39.8
4β	1.70, ddd (15.2, 3.0, 3.0)	
5	-	75.4
6α	1.66, m	37.8
6β	2.31, m	
7α	1.33, m	25.3
7β	2.11, m	
8	1.54, m	43.2
9	1.60	43.5
10	-	58.5
11α	1.59	22.9
11β	1.45, m	
12α	1.38, m	41.2
12β	1.49, m	
13	-	49.3
14	-	85.3
15α	1.65, dd (13.0, 9.0)	32.7
15β	2.02, ddd (12.8, 9.8, 9.8)	
16α	2.17, ddd (12.5, 9.5, 9.5)	29.6
16β	1.73, m	
17	2.53, dd (9.8, 6.6)	52.0
18	0.65, s	17.0
19	10.13, s	209.4
20	-	124.8
21	7.41, dd (2.6, 1.1)	150.6
22	7.96, dd (9.7, 2.6)	149.2
23	6.27, dd (9.7, 1.0)	115.5
24	-	164.7

[a] Overlapped signals were reported without designating multiplicity.
